# Neural network potentials for chemistry: concepts, applications and prospects

**DOI:** 10.1039/d2dd00102k

**Published:** 2022-12-21

**Authors:** Silvan Käser, Luis Itza Vazquez-Salazar, Markus Meuwly, Kai Töpfer

**Affiliations:** a Department of Chemistry, University of Basel Klingelbergstrasse 80 CH-4056 Basel Switzerland m.meuwly@unibas.ch kai.toepfer@unibas.ch

## Abstract

Artificial Neural Networks (NN) are already heavily involved in methods and applications for frequent tasks in the field of computational chemistry such as representation of potential energy surfaces (PES) and spectroscopic predictions. This perspective provides an overview of the foundations of neural network-based full-dimensional potential energy surfaces, their architectures, underlying concepts, their representation and applications to chemical systems. Methods for data generation and training procedures for PES construction are discussed and means for error assessment and refinement through transfer learning are presented. A selection of recent results illustrates the latest improvements regarding accuracy of PES representations and system size limitations in dynamics simulations, but also NN application enabling direct prediction of physical results without dynamics simulations. The aim is to provide an overview for the current state-of-the-art NN approaches in computational chemistry and also to point out the current challenges in enhancing reliability and applicability of NN methods on a larger scale.

## Abstract 2

Artificial Neural Networks (NN) are already heavily involved in methods and applications for frequent tasks in the field of computational chemistry such as representation of potential energy surfaces (PES) and spectroscopic predictions.

## Graphical abstract

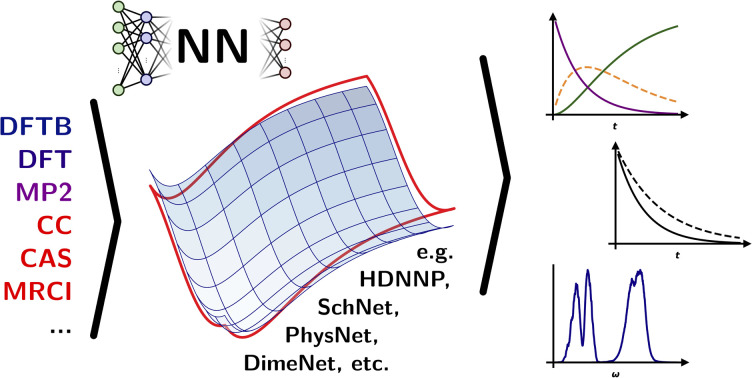

## 1 Introduction

The *in silico* modeling of chemical and biological processes at a molecular level is of central importance in today's research and will be crucial for future challenges of mankind.^1^ The modeling often requires a trade-off between accuracy and computational cost: quantum chemical calculations (*e.g. ab initio* molecular dynamics), at a high level of theory, can be very accurate but also come at a high computational cost rendering the approach impractical except for rather small molecules. Empirical force fields, on the other hand, provide a computationally advantageous approach that scales well with system size but the possibility to carry out quantitative studies is limited due to the assumptions underlying their formulation. Thus, computationally efficient and accurate modelling techniques are required for quantitative molecular simulations.^2^

In this regard, Machine Learning (ML) techniques have emerged as a powerful tool to satisfy such demands for force field models which are limited, in principle, by the accuracy of *ab initio* methods and allow an efficiency approaching that of empirical force fields.^3^ Motivated by the advances in computational chemistry techniques and the continuous growth of the performance of computer hardware (Moore's law^4^), ML is becoming a daily tool for modeling molecules and materials. By definition, ML methods are data-driven algorithms based on statistical learning theory with the aim of generating numerical methods that generalize to new data, not used in the learning process.^5,6^ This capability renders ML methods highly appealing for modelling molecular systems. It even reaches levels where some authors believe that the use of ML techniques will constitute the “fourth paradigm of science”,^7^ bridging the gap from atomic-scale molecular properties towards macroscopic properties of materials^8,9^ and one of the drivers for a revolution of the simulation techniques of matter.^10^ The enthusiasm is reflected in the appearance of an extensive number of ML models and their application in computational chemistry.

Some of the most important publications have focused on the study of potential energy surfaces (PESs), which contain all the information about the many-body interactions of a molecular system including stable and metastable structures.^11^ At the same time, it is possible to extract a considerable amount of information from PESs including the atomic forces driving the dynamics of molecular systems, reactions and structural transitions, and atomic vibrations.^12^ Additionally, it has been proposed that the chemical information contained in a chemical bond, therefore in the PES, can help in the exploration of chemical space.^13^ In a recent work,^14^ it was found that the exploration of chemical space can be improved by adding adequate information from the configurational space represented by the PES.

Over the past several decades several ML-based methods have been used to represent continuous PESs.^3,15–17^ While a number of those are briefly mentioned below, the focus of the present work is on NN-based approaches. Kernel-based methods provide an efficient solution to highly non-linear optimization problems^17^ by finding a representation of the problem which encodes the distribution of the data in a complete, unique and efficient way.^18^ There is a large number of possible representations of chemical space that can be used in kernel methods. Examples include Coulomb Matrices,^19^ Bag of Bonds (BoB),^20^ Histograms of Distance, Angles and Dihedrals (HDAD),^21^ Spectrum of London and Axilrod–Teller–Muto (SLATM),^22^ Faber–Christensen–Huang–von Lilienfeld (FCHL)^23^ and Smooth Overlap of Atomic Positions (SOAP).^24^ A comprehensive review of representations for kernel and non-kernel methods can be found in ref. 25. It should be noted that variations of kernel methods, such as for Gaussian processes^26^ which assume a Bayesian/probabilistic point of view for the solution of the problem or the reproducing kernel Hilbert space (RKHS) method^27,28^ which uses polynomials as support functions have been extensively discussed in the literature. While the remainder of the perspective is mainly dedicated to NN-based approaches, many alternative interpolation and representation methods for PES construction exist. These include, *e.g.* modified Shepard interpolation,^29^ (interpolative) moving least-squares,^30–32^ permutationally invariant polynomial (PIP) PESs by least-squares fitting,^33^ or least absolute shrinkage and selection operator (LASSO) constrained least-squares.^34^ Several of these approaches have been recently described, reviewed and compared.^3,35,36^

NNs are inspired by the biological model of the intricate networks formed by the brain and how information is passed.^37^ The ideas underlying NNs date back to 1960 when “the perceptron” was presented by Rosenblatt.^38^ However, computational and theoretical limitations inhibited the development of NNs.^39,40^ It was not until 1970 with the development of the automatic differentiation and the introduction of backpropagation^41^ that NN models continued to develop. Still, large scale applications were rare until the beginning of the 21st century when considerably more powerful computer hardware became available. In chemistry, the application of NN models dates back to 1990s with first applications in analytical and medicinal chemistry.^42,43^ Regarding PES representation, the first application of NNs can be tracked back to the same decade.^44,45^ Nowadays, NNs are the most common ones from the field of ML models for the use in chemistry-related applications that are focused on the generation and study of PESs. Some examples of popular NN-based schemes for PES fitting include the High Dimensional Neural Network (HDNN) method,^46,47^ Deep Tensor Neural Network (DTNN),^48^ SchNet,^49^ ANI,^50^ or PhysNet,^51^ among others.

The purpose of the present perspective is to provide a birds-eye view and an outlook into the conception, generation and use of NN based PESs for the exploration of chemical systems. Additionally, we will present some of the current challenges in the development and application of NN models for the study of PESs. The remainder of the present work is structured as follows. A brief introduction to the theoretical background of PESs and NNs is provided in Section 2. Section 3 discusses existing NN architectures with emphasis on structural information and current developments in the field. Section 4 describes the construction of a PES from the initial sampling to the validation and refinement of the generated models and Section 5 discusses knowledge transfer that allows obtaining PESs at high levels of theory with less data. Selected applications for chemical systems showcasing the concepts introduced and including NN models in established atomistic dynamics models are described in Section 6. Applications of NN models that skip dynamics simulation to predict physical observables are shown in Section 7. Section 8 describes some of the current challenges that we consider critical for the development and enhancement of the current models and the field in general, followed by a short conclusion.

## 2 Theoretical background

This section introduces the concept of PESs, the principles underlying NNs, their building blocks, such as dense layers and activation functions. A more in-depth overview of descriptors for chemical structures and representative examples of frequently used neural network potentials (NNPs) is given in the next section. In terms of nomenclature, italic symbols denote scalars or functions and bold symbols are *n* – dimensional tensors (*n* ≥ 1) with the special case of a one-dimensional spatial vector (*e.g.* position or distance) denoted as italic symbol with vector arrow.

### 2.1 Potential energy surfaces

The energetics of a molecular system can be described by solving the electronic Schrödinger Equation (SE). Unfortunately, the SE can only be solved exactly for simple, single-electron atomic systems. In order to obtain solutions for many-electron systems, it is necessary to introduce approximations. The Born–Oppenheimer approximation (BOA),^52^ also called the most important approximation in quantum chemistry,^53^ assumes that the coupling between the nuclear and electronic motion can be neglected because the mass of the nuclei is several orders of magnitude larger than the mass of the electrons. Under this assumption, it is possible to rewrite the total wavefunction *Ψ*, which is a solution of the SE, as the product of a nuclear wavefunction *χ*(**R**) with nuclear positions **R** and the electronic wavefunction *ψ*(**r**;**R**) with electron coordinates **r** for a fixed configuration of nuclear positions

1 *Ψ*(**r**,**R**) = *ψ*(**r**;**R**)·*χ*(**R**).

As a consequence, the electronic wavefunction can be obtained by solving the electronic time-independent SE:2
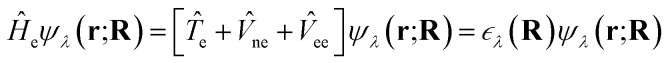
Here, *Ĥ*_e_ is the electronic (spin-free) Hamiltonian describing the kinetic energy of the electrons *T̂*_e_, the Coulomb interaction between the nuclear and electron charges *V̂*_ne_ and the electron–electron interaction *V̂*_ee_. The solution is the electronic wavefunction *ψ*_*λ*_ and electronic energy 
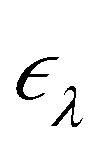
 for the electronic state *λ*. The so-called adiabatic PES of an atomic system *E*^BO^_*λ*_(**R**) in electronic state *λ* constitutes an effective potential for the nuclear dynamics. It is obtained by the sum of the Coulomb repulsion *V*_nn_ between the nuclei with nuclear charge *Z*_*i*_ for the total number of atoms *N*, and the respective electronic energy at the associated nuclear positions.^54^3
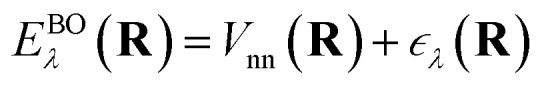


**Fig. 1:**
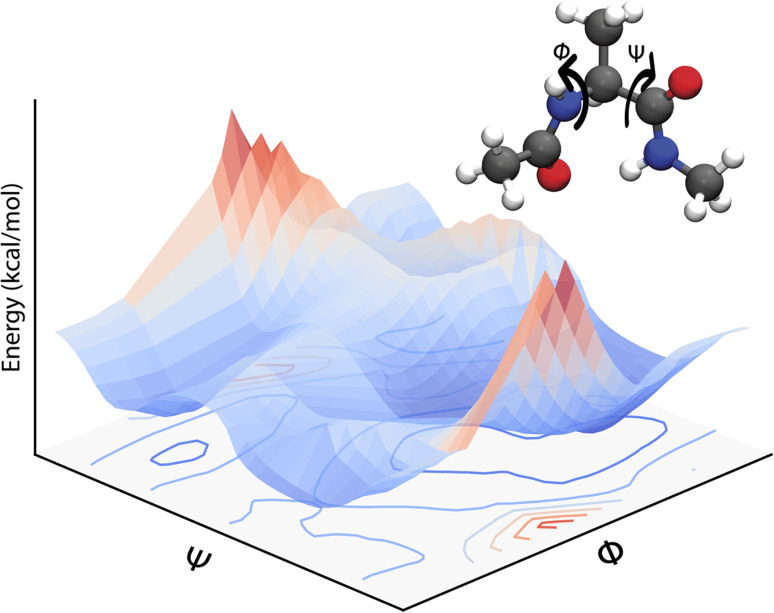
A two-dimensional PES for the dialanine molecule calculated at the MP2 level with the 6-31G** basis set along dihedral angles *Φ* and *Ψ*. A representation of the molecule (ball and stick) indicating the dihedral angles (*Φ*, *Ψ*) calculated is given as well. The bottom gives the projection of the 2D PES.

Eqn (3) defines a PES as a (3*N* − 6) – dimensional function that can be approximated as an analytical function which is, however, a challenging task. Often, one can only report low-dimensional cuts of such high-dimensional hypersurfaces and one example is shown in Fig. 1. Alternatively, eqn (3) suggests that there should be a mapping between the total electronic energy of a molecular system and the combination of position of the nuclei and the set of nuclear charges {*Z*_*i*_}^*N*^_*i*=1_. This is the starting point for a ML-based approach described in the following.

PESs lie at the heart of computational chemistry.^55^ From the relationship between structure and potential energy *E*, it is possible to derive many molecular properties by taking derivatives with respect to a perturbation such as atomic positions **R**, an external electric 
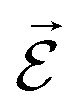
 or magnetic field 
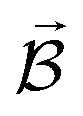
, which require additional coupling terms in the Hamiltonian and an analytical representation of the PES.^54^ Following this, a general response property takes the form4
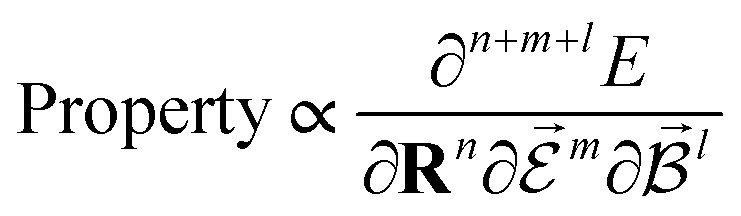
where *n*, *m*, *l* indicate the order of the derivative with respect to the perturbation. Derivatives of eqn (4) provide, *e.g.*, the forces **F** = −∂*E*/∂**R** that constitute the foundation of MD simulations and structure optimization schemes. The second derivatives ∂^2^*E*/∂**R**^2^ gives access to the Hessian matrix from which the harmonic frequencies of molecular vibrations can be obtained. Other properties such as the dipole moment 
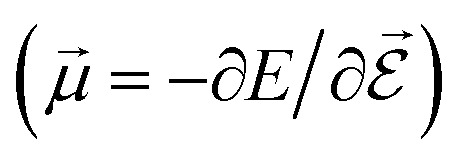
 or the molecular polarizability 
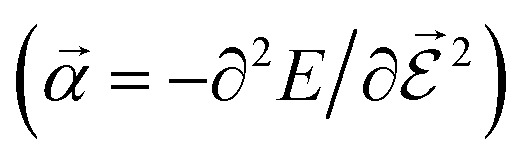
 are directly related to experimental observables such as the Infrared (IR) or Raman spectra.^56^ Mixed derivatives also provide IR absorption intensities 
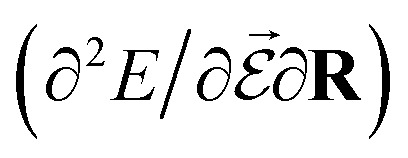
 or the optical rotation in circular dichroism 
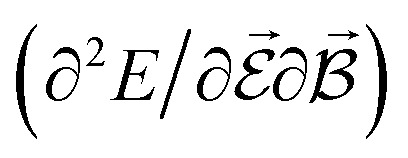
.

Given the versatility and usefulness of PESs, a wealth of approaches to construct PESs have been designed over the years and new ML schemes are proposed with high frequency. Especially NNs have been shown to be general function approximators^57,58^ by the universal approximation theorem^59^ and hence seem particularly useful to learn intricate relationships such as the PES or even external perturbations.

### 2.2 Artificial neural networks

Artificial NNs (NNs, henceforth) represent a family of computer algorithms and form a subgroup of ML. Nowadays, NNs are applied in diverse areas including, among others, health care,^60^ medical imaging,^61^ self-driving cars,^62^ high-energy physics,^63^ particle physics and cosmology,^64^ genetics,^65^ chemical discovery,^66^ reaction planning.^67,68^

**Fig. 2:**
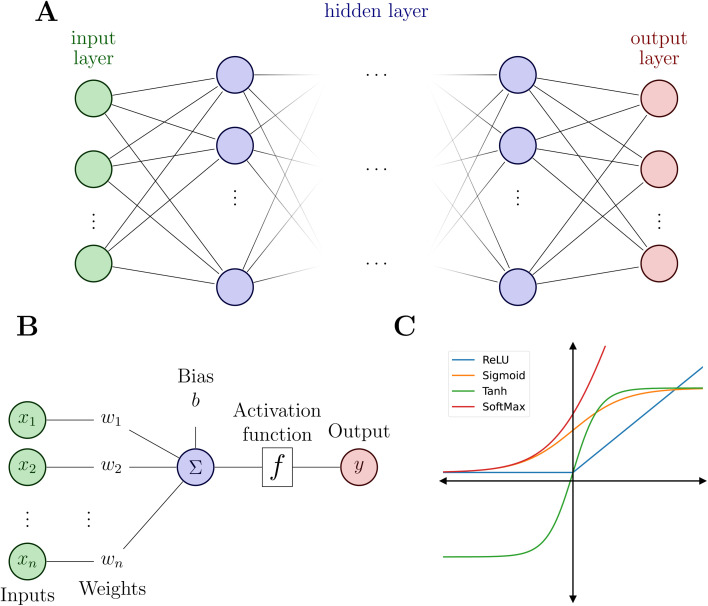
Neural network and its building blocks. (A) Schematic of a NN model with an input layer (green), *N* hidden layers (blue) and an output layer (red). (B) Illustration of a node inside the hidden layers. Bottom right (C): examples of common activation functions.

Typically, a NN consists of an input layer, a predefined number of hidden layers and an output layer (see Fig. 2A). Deep NNs comprise a larger number of hidden layers while a NN with only one or two hidden layers is a shallow NN. Each layer contains a defined number of nodes (or neurons) that connect to the nodes of the following layer and each connection is associated with weights and biases.

The elementary units of NNs are so-called dense layers, which linearly transform an input vector **x** to an output vector **y** according to

5 **y** = **Wx** + **b**.

Here, **W** = {*w*_*ij*_}^*N*,*M*^_*i*,*j*=1_ and **b** = {*b*_*i*_}^*N*^_*i*=1_ are the weights (a matrix) and biases (a vector),^3^*M* is the dimension of the input and *N* the number of nodes. The combination of a dense layer with a nonlinear activation function (Fig. 2B) transforms the input **x** to an output **y** that serves as “input” to the following (hidden) layer.

6 **h**_*i*_ = *σ*(**W**_*i*_**x** + **b**_*i*_)

Modelling non-linear relationships requires the combination of at least two dense layers with an activation function *σ* according to

7 **y** = **W**_*i*+1_*σ*(**W**_*i*_**x** + **b**_*i*_) + **b**_*i*+1_ = **W**_*i*+1_**h**_*i*_ + **b**_*i*+1_

While such shallow architectures are in principle capable of modelling any functional relationship, deeper variants thereof are usually preferred due to improved performance and parameter-efficiency.^69–72^ The functional form of the NN is characterized by the number of layers *L* and number of nodes *N* in a given layer. With increasing *L* and *N* the functional form becomes more flexible, however, overfitting requires careful attention since the obtained form has no underlying physical meaning.^73^ A fully connected deep NN is given by the following relation8
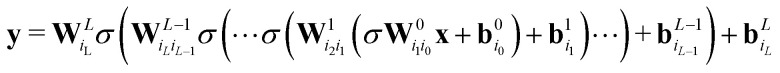
which is usually followed by a linear transformation in the final output layer to yield the prediction **y**_*L*+1_. If the NN is used to construct a PES, a chemical descriptor **x** is mapped onto one or multiple scalar values **y** = {*V*}, which are the energies of one or several electronic states for an atomic configuration.

As mentioned above, the flexibility and power of a NN is related to the number of layers and nodes but the ability to obtain highly non-linear relationships between inputs and outputs is a consequence of the use of appropriate activation functions (Fig. 2C). Activation functions usually satisfy particular mathematical properties, including differentiability (crucial for computing forces or vibrational frequencies)^74^ and smoothness, that simplifies the optimization of the model and increasing the quality of the prediction of energy and forces.^75^

Besides the architecture of a NN, the actual training (or “learning”) step is important, too. Training comprises the parameter fitting process of the weights and biases to match the prediction **y**(**x**) to the reference results **t** for a set of *N*_data_ data points. The accuracy of the fit is measured by monitoring a loss function 
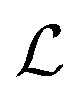
 which has the general form:^75^9
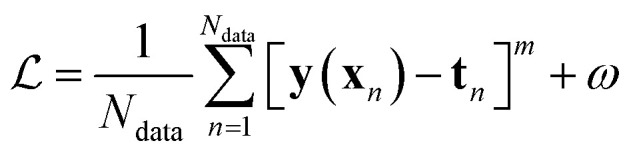


The value of *m* in eqn (9) mostly takes the value *m* = 1 or 2 (*L*_1_ or *L*_2_ norm) and *ω* can be a regularization term that helps to improve the generalizability of the model and to prevent overfitting (*i.e.* the model is fitted perfectly against training data losing generalizability). Different loss functions for fitting NNs can be used as well.^76^ In general, the loss function is highly nonlinear and is minimized iteratively by a gradient descent algorithm which, preferably, can find the best solution despite potentially many local minima.^56^ For PES fitting, convergence behaviour and accuracy can be improved by including additional information such as atomic forces or dipole moments (or other properties of the system) in the loss function.

## 3 Neural networks for potential energy surfaces

The use of NNs to represent PESs of molecular systems started in the 1990s. However, initially it was only possible to include a few degrees of freedom.^42,77–80^ Applicability and transferability of NNs to larger systems and with different system compositions were improved by the approach proposed by Behler and Parrinello who decomposed the total energy of a system into atomic contributions^46^10
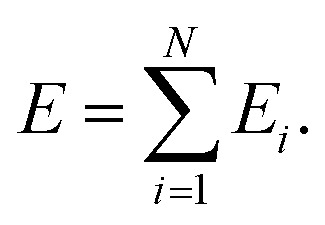
Here, *N* is the total number of atoms and *E*_*i*_ is the energy of atom *i* that can be predicted by one or multiple NNs (*e.g.* one for each atomic element). The inputs are local, atom-centered descriptors that encode the local chemical environment around atom *i*. Rooted in eqn (10), the so-called high-dimensional NNP (HDNNP),^46,81^ was introduced and followed by further models.^47,48,50,51,82–96^ It is important to note that most of the commonly used models are based on the decomposition of the energy in atomic contributions, although models that represent the energy as the sum of bond energies have also been proposed.^97–99^ In the following, we will focus on NNs that decompose the potential energy into atomic contributions.

### 3.1 Descriptors

All NNs are based on a local representation of the chemical environment to correctly predict the reference data.^24,100–103^ Such representations require descriptors that, most importantly, are (i) *invariant* with respect to transformations including translation, rotation and permutation of same elements, (ii) *unique* by showing changes when transformation that modify the predicted property are applied and (iii) *continuous* and *differentiable* with respect to the atomic coordinates to determine forces for molecular simulations.^101,103^ Based on the type of local representation that incorporates all the conditions above, NNPs can be classified into two major categories: those with predefined and those with learnable descriptors.^15^

#### 3.1.1 Predefined descriptors

Encoding the atomic environment by descriptors that fulfill the previously described characteristics has been a challenge since the early beginnings of the development of NN models and it is still an area of active development. Some of the requirements for a “good” descriptor can be matched with simple transformations of the Cartesian atom positions. For example, rotational and translational invariance can be obtained by using internal coordinates.^11,104^ However, permutational invariance is more difficult to incorporate. A solution to this problem is the use of PIPs^33^ as input for a NNP, which are still extensively used for small molecule PESs.^105–110^ Other solutions are based on using symmetrized input coordinates or symmetry incorporated in the NN.^104^

A better solution to the problems described above was found with predefined descriptors introduced by Behler and Parrinello in 2007 with the development of the HDNNP.^46,47,50,81,84,90,92,93^ These descriptors, termed atom-centered symmetry functions (ACSF)^81,111^ or variations^50,91^ thereof are the prevalent predefined descriptors for NNPs in the literature.

Originally, the local chemical environment of atom *i* is encoded by sets of radial- and angular-type symmetry functions *G*^rad^_*i*_ and *G*^ang^_*i*_ for each element or element combination of atoms *j* and *k* individually. A modified version of Gastegger and coworkers, on the other hand, combines them linearly with a weighting factor depending on the respective atoms' element number *Z*_*j*_ and *Z*_*k*_.^112^11
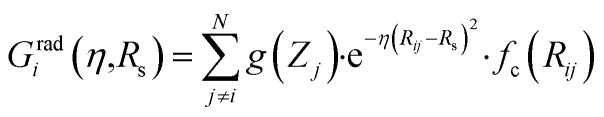
12
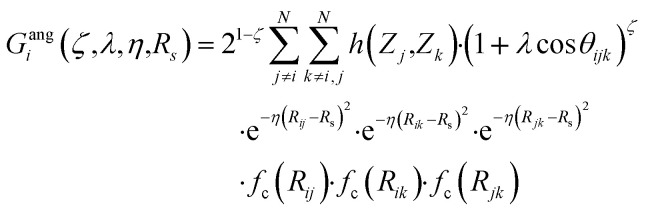


In this version of weighted ACSF (wACSF) *R*_*ij*_, *R*_*ik*_, *R*_*jk*_ are pair distances and the angle *θ*_*ijk*_ is defined between the vectors 
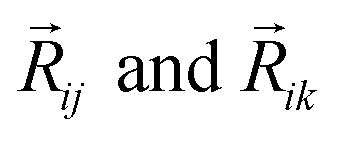
. The contributions to the symmetry function are limited by the cutoff function *f*_c_(*R*) which monotonically decrease from 1 to 0 at the cutoff separation *R*_c_. The parameter *λ* ∈ { −1,1} determines the maxima of the cosine term at *θ*_*ijk*_ = 0° or 180°. The resolution and size of the descriptor are determined by the choice and number of combinations of hyperparameters *η* and *R*_s_ for the radial symmetry functions *G*^rad^_*i*_ as well as *ζ* and *η* for the angular symmetry functions *G*^ang^_*i*_. The functions *g*(*Z*_*j*_) and *h*(*Z*_*j*_,*Z*_*k*_) are the element-dependent weighting functions for which even simple expressions such as *g*(*Z*_*j*_) = *Z*_*j*_ and *h*(*Z*_*j*_,*Z*_*k*_) = *Z*_*j*_*Z*_*k*_ yielded satisfactory results.^112^

Regarding the ACSF representation, each descriptor is a vector for which the length depends on combinations of the sizes of respective hyperparameters *η*, *R*_s_ and *ζ* with size *N*_par_ but also the number of different chemical elements *N*_el_ in the atomic system. These are *N*_par_·*N*_el_ for radial-type and *N*_par_·*N*_el_(*N*_el_ + 1)/2 for angular-type symmetry functions. The size of the radial- and angular-type wACSF simply scales by the respective combination of the hyperparameters. HDNNPs with descriptor sizes of 32 wACSFs, 220 ACSFs and 35 ACSFs were trained using the energies of the molecules in the QM9 database with up to five elements. The mean absolute error of the validation and test set is reported even lower for the model with wACSFs (1.84 and 1.83 kcal mol^−1^, respectively) than the 220 ACSFs (2.49 and 2.39 kcal mol^−1^) and 35 ACSFs (7.57 and 7.40 kcal mol^−1^).^112^

ACSFs commonly apply expensive trigonometric cutoff functions but computationally much cheaper polynomial cutoff functions can be designed for the same functionality.^113^ Further improvement in the performance is achieved by replacing the exponential function and cosine in radial- and angular-type symmetry function with dedicated polynomials with essentially no loss in accuracy.^114^ The speedup is shown by MD simulations of 360 water molecules using a HDNNP that performs about 1.8 times faster with polynomial symmetry and cutoff functions than with the original ACSFs.^114^

Another type of fixed descriptors was introduced by E and coworkers in their Deep Potential (DP) model.^82,115^ These are based on the construction of a local coordinate frame which assures the required invariances. Once the positions of the atoms are transformed by a translation and rotational matrix, the local coordinates can be used to construct the descriptor based on radial and/or angular information. However, this descriptor cannot ensure smoothness because of the uncertainty in the choice of the local frame that can lead to discontinuities.^116^ E and coworkers proposed the Deep Potential-Smooth Edition (DP-SE) model^117^ to solve the mentioned issue by enforcing continuity of the descriptor by multiplying the local coordinate system with a continuous and differentiable function and modifying the embedding matrix to recover two-body and three-body terms of the descriptor.^116^

In addition to the ACSF functions and the DP descriptor, there are other descriptors that utilize the concept of neighbourhood density functions.^118,119^ For this type of descriptors the information about the local environment of atom *i* up to a cutoff radius is represented by a density function *ρ*(**R**_*i*_) depending on the nuclear charge *Z*_*j*_ and position **R**_*j*_ of neighbouring atoms *j*.13
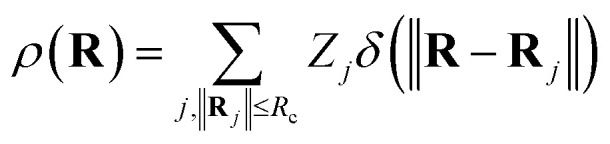
Here, *δ* is the Dirac delta function. In order to use this function in a NNP, it is necessary to expand *ρ*(**R**_i_) in a basis set of fixed dimension. For Gaussian-type basis functions, the ACSF functions are obtained.^118^ Other interesting expansions include the use of Zernike basis sets in which radial basis functions and spherical harmonics polynomials are used.^119^

A major problem of using predefined descriptors is that it requires a certain degree of knowledge to define the hyperparameters appropriately.^47,50,81,84,90,92,93^ Even though some of the hyperparameters can be optimized during the training as well,^83,91^ a poor choice of hyperparameters can lead to limited resolution of certain atomic displacements with quasi-constant descriptors and degenerate values of the predicted energy for different geometrical structures.^120,121^ The disadvantages of fixed descriptors motivated the emergence of NNPs which directly learn a suitable representation of atomic positions and element types.^3,74^

#### 3.1.2 Learnable descriptors

The concept of learnable descriptors originates from graph neural networks.^122^ In general, atoms are regarded as nodes (not to be confused with nodes of NN layers), each associated with a feature vector, which are connected to their neighbouring atoms within a cutoff sphere by so-called edges. Information between the nodes is passed along the edges over multiple iterations to encode the necessary chemical interaction.

**Fig. 3:**
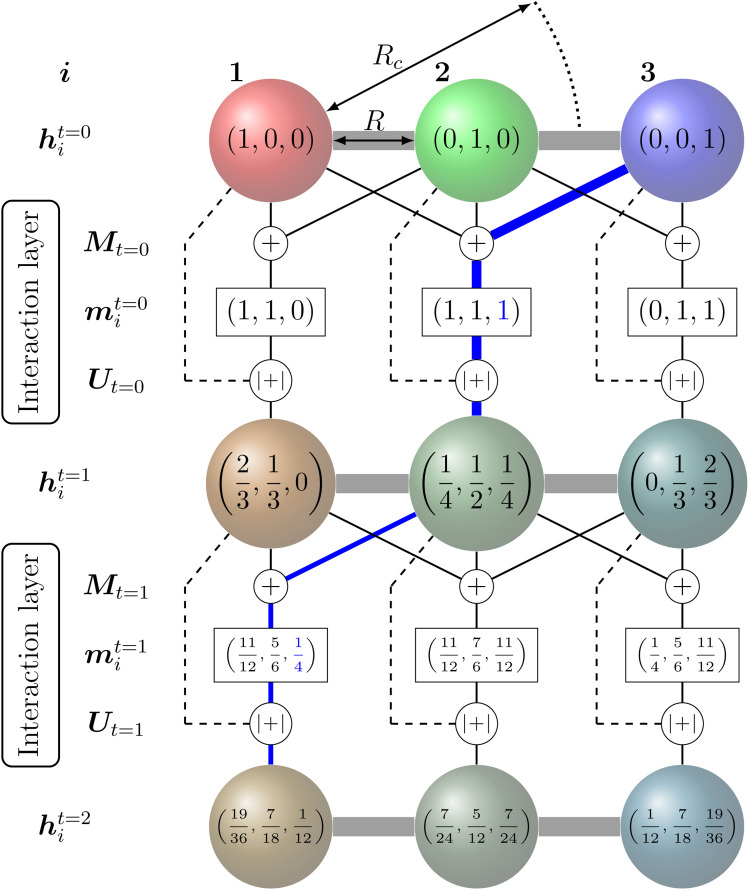
Message-passing principle visualized on a chain of three nodes with initial feature vectors *h*_*i*_^*t*=0^ representing the colour fraction red, green, blue on the mixed colour of node *i*. The message operation *M*_*t*_ corresponds to the addition of the feature vectors within in cutoff range and the update operation *U*_*t*_ corresponds to an addition of *h*_*i*_^*t*+1^ = *h*_*i*_^*t*^ + *m*_*i*_^*t*^ and scaling that sum{*h*_*i*_^*t*+1^} = 1. Although it is outside the cutoff radius *R*_c_, after two iterations the feature vector of node 1 (*h*_1_^*t*^=2) contains a fraction (information) of the initial feature vector from node 3 (visualized by the blue coloured path).

The feature vectors of each node with length *N*_f_ are randomly initialized as a function of the atoms' nuclear charge, that is iteratively updated by a message vector encrypting structural information and feature vectors of the atoms within a cutoff sphere by passing through interaction layers which ensure the required invariances. Fig. 3 visualizes the message passing principle on a linear chain of nodes (atoms) with distance *R*, where the feature vector *h*_*i*_^*t*^ at each iteration step *t* corresponds to the ratio of the colours red, green and blue to the mixed colour. In each interaction layer, the feature vectors of node *i* and connected nodes within cutoff range *R*_c_ are combined by a message function *M*_*t*_ (addition) to the message vector *m*_*i*_^*t*^. Note that this message function does not encode distances *R*. The message vector *m*_*i*_^*t*^ is combined with the feature vector *h*_*i*_^*t*^ by an update function *U*_*t*_ (addition and scaling to linear sum of 1) to form a refined feature vector *h*_*i*_^*t*+1^ that contains information of the surrounding nodes. Message and update functions usually include the transformation of feature with update vectors by a NN. For an iteration step *t* > 1, this approach allows that information from nodes that are outside of the cutoff range can still be incorporated in a feature vector of a given node *i* indirectly. This means that for the case illustrated in Fig. 3, the feature vector *h*_1_^*t*=2^ of node 1 contains a fraction of blue colour after two iterations 
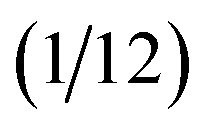
 that is passed from node 3 *via* node 2.

Many of the more recently developed NNPs^48,51,85–89,94–96^ apply such atom-wise feature vector approaches and are called message-passing NNs (MPNNs).^123,124^ Depending on the MPNN model, the atomic feature vectors of either the final iteration or each iteration are passed to a specific NN and transformed into the desired quantity (*e.g.* energy).

Feature vectors with higher number of elements *N*_f_ and more complex message and update functions including bond distance and direction dependencies allow higher resolution of the structural encoding. In common NNPs, the number of elements in the feature vectors *N*_f_ range from about 64 to 128 per element. A larger number might increase the risk of overfitting.^86^ Similarly, a larger number of message passing iterations improves the representation of the structural features but the potential energy accuracy usually shows sufficient saturation after three iteration (*t* = 3).^48,85–87,94^

### 3.2 Architectures

Given that the field of NNPs is very active, it is impossible to describe all the available NN architectures. Hence this section is not a comprehensive review of all possible architectures but rather a more history-guided view of architectures and what functionalities were included in subsequent development steps.

Initial models use NNs as a method for the fitting of PES only (no forces).^125^ These models were limited to small molecules in gas phase and were fitted to energies of *ab initio* calculations *via* a many-body expansion^126^ or a high-dimensional model representation.^127^ Therefore, these models take energies and positions to predict coefficients for a defined functional form. These models already achieved spectroscopic accuracy for small molecules.^12^

The introduction of the HDNNP with the concept of decomposing the molecular energy into atomic contributions (eqn (10)) changes the paradigm of NNPs. A new challenge was encoding the local environment information sufficiently well for an accurate energy prediction that lead to the two main approaches of predefined or learnable descriptors. The main development of NN architectures with predefined descriptors goes towards more sophisticated descriptors to encode atom-centered properties which are then provided to standard fully-connected feed-forward NNs.^128^ NN architectures with learnable descriptors and the MPNN approach differ in their message and update functions within an interactions layer.

The first MPNN proposed was the deep tensor neural network (DTNN)^48^ by Schütt and coworkers that had been further improved into the, to this day, popular SchNet model.^85^ An interaction layer in SchNet includes so called continuous-filter convolutional layers that have already been used in image or sound processing.^85^ A combination of the popular predefined ACSF descriptors and learnable ones was proposed by Isayev and coworkers and their atoms-in-molecule NN model (AIMNet).^87^ Modified ACSF descriptors from the ANI architecture were used for initialization of atomic structure feature vectors, combined with atomic information feature vectors and passed through the interaction layer.

Although these models already achieve good accuracy, long range interactions between chemical compounds can only contribute to the total energy if the information is included in or passed to the descriptor by a sufficiently long cutoff range *R*_c_. Systems with strong electrostatic interactions, especially with highly polar or ionic chemical species, requires larger cutoffs but at the cost of higher computational demand.^51^ One solution is to add a Coulomb term to the atomic energy contributions which includes electrostatic interactions between atomic charges *q* predicted by the NN model.14
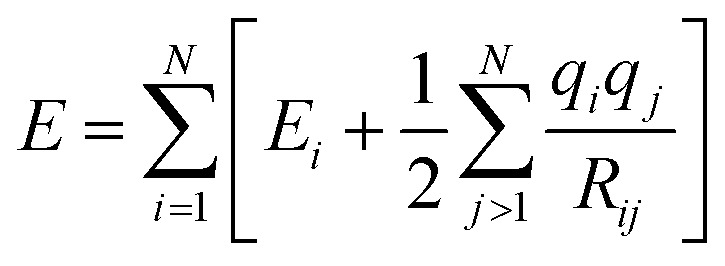


The earliest NN model using eqn (14) was introduced by Artrith and Behler in 2011 that trains a separate NN with reference charges from a Hirshfeld population analysis.^90^ Another approach is applied by the TensorMol model that predict atom charges by fitting the *ab initio* and physically determinable molecular dipole moment to the predicted one computed by the atom charges.^91^

Additional physically motivated interactions, such as dispersion interactions, were also included in the TensorMol model but have been employed in PhysNet, too. PhysNet is based on the MPNN architecture and was developed by Unke and Meuwly.^51^ It does not only add an energy contribution from the DFT-D3 dispersion correction scheme^129^ but also modifies eqn (14) by applying a damping function that smoothly damps Coulomb interactions for small atom distances to avoid singularities15
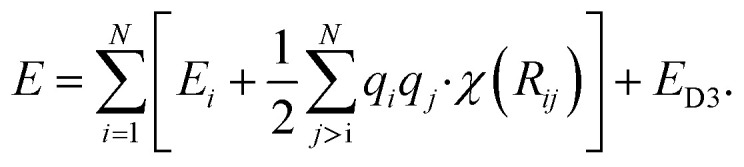


*E*_D3_ is the DFT-D3 dispersion correction and the damping function *χ*(**R**_*ij*_) is defined as:16
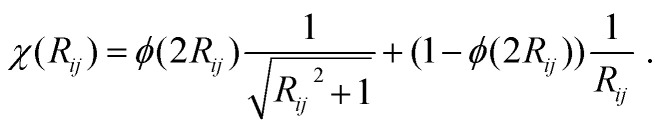


A continuous behaviour is ensured by the cutoff function *ϕ*(*R*_*ij*_).

Although adding a Coulomb term to NNPs improves the description of long range interactions while the atomic charges still depend on the local chemical environment.^104^ However, chemical systems are inherently non-local. Therefore, the approximation breaks down for systems with changes in the total charge state (*i.e.* ionization, protonation or deprotonation), electronic delocalization or spin density rearrangements.^89^ These effects are difficult to capture with NN architectures which model changes in the atom charges by local perturbations.

The most recent generation of NNPs addresses the problem of non-local charge transfer by using different strategies. The first work dedicated to the issue of charge equilibration was the “charge equilibration *via* NN technique” (CENT) developed by Ghasemi and coworkers.^92^ The CENT algorithm equilibrates the charge density to minimize the electrostatic energy which depends on environment-dependent atomic electronegativity and hardness besides the charge–charge interaction. Inspired by CENT, Behler and coworkers introduced their fourth generation HDNNP (4G-HDNNP) model where NNs are trained to predict environment-dependent atomic electronegativities (constant element-specific hardness) and the charge equilibration yields the reference atomic charges.^47^ In a second training step, NNs provided with ACSFs and the atomic charge information are trained to predict the short-range atomic energy contributions which sum up with the electrostatics to the correct reference energy and forces.

SpookyNet is a MPNN model and introduced by Unke and coworkers that treats the problem of non-locality by creating an embedding for charges and spin.^89^ It is capable to predict molecular systems with different spins and charged states as provided in the reference data set within one single model. The general idea of predicting PESs of chemical systems for different electronic states and their coupling strength within one model is an area of active research.^130^ One model in this direction that can be mentioned is SchNarc^131^ that combines the SchNet model with the surface hopping including arbitrary couplings (SHARC)^132^ code.

So far, we have been reporting the effort to improve the models accuracy by introducing more physically motivated interactions. However, current developments for MPNNs focus on passing spatial directions between atoms to the NN that allow the prediction of atom-centered tensorial properties such as atomic polarizability.^94,133,134^ Providing solely distance information inherently ensures translational and rotational invariance for atom-centered scalar properties (predictions do not change with respect to, *e.g.*, rotation of the molecule). The challenge with directional information is rotational equivariance which means that predicted atom-centered directional properties 
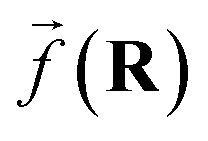
 keep its amplitude but change in direction equivalent to a rotation 
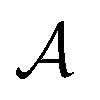
 of the molecular coordinates **R**.17
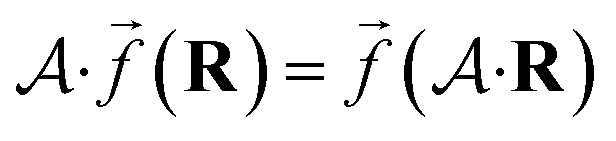


MPNNs that encode directional information (directional message passing) and fulfill eqn (17) are called equivariant NNs (ENNs).^135,136^

ENNs have been proven to be data-efficient and capable of providing better predictions of tensorial quantities (*i.e.* dipole, quadrupole moments) than invariant models. ENN models with different modifications were suggested to include directional information and assure equivariance. Some of them are PaiNN,^94^ NeuqIP,^96^ and NewtonNet.^137^ Still one of the best performing ENNs on the QM9 data set is DimeNet, where rotational equivariance is achieved by representing the local chemical environment of an atom by spherical 2D Fourier–Bessel basis with radial basis functions to represent bond distances and spherical basis functions to represent angles between bonds towards neighbouring atoms.^133^

Many NN potentials are often additionally designed for application on periodic systems including solids and crystals,^49^ or were updated to support periodicity.^138^ Others are specifically designed to train on reference data to predict formation energy, lattice parameters of the unit cells and other material properties directly from the structural fingerprint.^139,140^ The application of ML (including NNs) to materials has been discussed in detail in recent reviews^141–143^ and is not further considered in the present work.

The field of NNs in computational chemistry has been and will continue to be steadily developed to improve the capability and accuracy in predicting reference data. In consequence, the selection of a model should be done based on the problem at hand, the availability of the code, its user friendliness, and the computational resources available. It might not be necessary to use the most sophisticated model if the task does not require that level of description. Most of the previously described architectures are based on open source NN frameworks like Tensorflow^144^ or PyTorch^145^ which open the possibility to modifications and enhancements of the described models.

## 4 Construction of PESs

The collection of reference structures is an essential step in constructing a molecular PES, especially since the underlying functional form of the potential is not based on physical laws and is inferred purely from reference data.^104^ Besides the unfavourable scaling of the configurational space with system size, the computational expense associated with a reference point is usually high and depends on the level of quantum chemical theory used. Thus, the number of expensive and non-trivial *ab initio* calculations needs to be restricted to a minimum and optimally covers the configurational space most important/representative (this is an open question in itself) to the problem at hand.^3,146^ Ultimately, the configurational space that is covered by the reference data set defines the boundaries of application of the NNP. Therefore, knowing the application(s) for which the PES will be used is essential when generating the data.

**Fig. 4:**
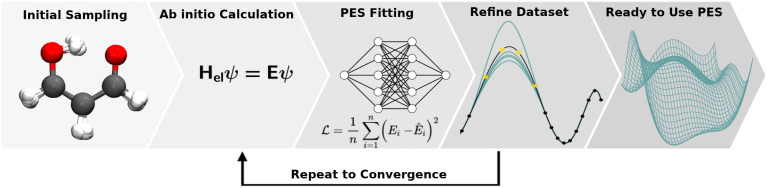
The process of PES generation: the configurational space of a chemical system (here malonaldehyde) is sampled to obtain an initial set of geometries. A quantum chemical *ab initio* calculation is carried out for each geometry to obtain reference data (including energies). After a NNP is fitted to the initial reference data set the resulting PES is validated thoroughly to find holes. New *ab initio* calculations are run for scarcely sampled regions and a new NNP is fitted. These steps are repeated until the PES has the required quality before the PES can be used to study the chemical system.

Reference data sets can be generated using a multitude of strategies which often requires the generation of an initial data set and refining it iteratively. This iterative process is illustrated in Fig. 4. Commonly employed strategies for structure sampling, which are often combined, will be described in the following. In addition to methods reviewed here, other possibilities include virtual reality sampling,^147–150^ Boltzmann machines^151^ or sampling based on the AMONS approach.^22^

### 4.1 Initial sampling

#### 4.1.1 *Ab Initio* MD

*Ab initio* MD (AIMD) constitutes an established means for generating reference data that samples a part of the configuration space of a chemical system.^104^ The temperature *T* (or the velocities that are drawn from a Maxwell–Boltzmann distribution corresponding to *T*) at which the simulation is run determines which part of a PES is sampled, how strongly the molecular geometries are distorted and whether or not reaction barriers are crossed. If the chemical system under investigation has multiple isomers, AIMD simulations can be run for all of them (partly) avoiding the need of running a long simulation that samples all isomers. Ideally, the sampling temperature *T* is chosen to be higher than the temperature at which the NNP is used. In other words, if the reference data set that was used to train a NNP was generated at *T* = 300 K the NNP should not be used to run simulations at *T* > 300 K because (most likely) configurations outside of the reference data set are visited leading to a breakdown of the NNP. Thus, running AIMD at a sufficiently high sampling temperature is needed to guarantee that the production runs do not enter the extrapolation regime, while the lower energy configurations are still sampled.^3^

The obvious disadvantage of running AIMD at the (final) level of theory at which the reference data set is generated is the high computational cost. This either limits the level of quantum chemical rigor or it limits the extent to which the configurational space can be sampled.^152^ Alternatively, configurations can be generated using sampling by proxy.^3^ This approach involves running AIMD at a lower level of theory to sample the PES and then perform single point *ab initio* calculations for a representative set of geometries at a higher level. This ideally requires that the topologies of the lower and the higher level of theory are similar to guarantee that the “correct” configurations are sampled. If the two PESs differ too much it is possible that the regions explored on the lower level PES do not correspond to relevant regions on the high level PES (which might happen if a force field is used to guide the sampling).^3,104^ As a consequence, the NNP could reach an extrapolation regime and exhibit a nonphysical behaviour.

Reactive chemical systems are usually associated with rare events. When NNPs are used to study reactive systems it is, thus, not sufficient to sample the reactant and product states since the reaction path (which is rarely visited in a simulation) needs to be part of the reference data set as well. TS regions can be sampled using AIMD by employing a scheme similar to umbrella sampling,^153^ in which geometries around the TS are sampled by harmonically biasing the molecule towards the TS.

A simulation technique that is related to MD simulations and can be used to generate configurations for the construction or refinement of a reference data set is metadynamics.^154^ Converse to ordinary MD, metadynamics uses history dependent biasing potentials to artificially increase the potential of visited regions on the PES and enhance the sampling of higher energy regions.

#### 4.1.2 Normal mode sampling

Normal mode sampling (NMS) was proposed to enable accelerated yet chemically/physically relevant sampling of a PES.^50^ As the name suggests, NMS uses the normal modes of vibration of a molecule to generate molecular geometries that cover configurational space at which single point calculations can be carried out at a desired level of theory. NMS is carried out as follows:^50^ (i) the molecule of interest is optimized at a desired level of theory (ii) normal mode coordinates *Q* = {*q*_*i*_} (*i.e.* eigenvectors of the mass-weighted Hessian) and corresponding force constants *K* = {*k*_*i*_} are determined (with *i* ∈ [1,*N*_f_ = 3*N* − 5] or *i* ∈ [1,*N*_f_ = 3*N* − 6], for linear and non-linear molecules, respectively) (iii) *N*_f_ uniformly distributed random numbers *c*_*i*_ with 
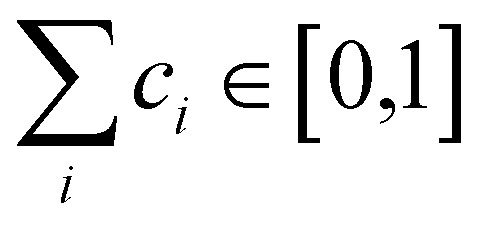
 are generated (iv) displacements for each normal mode are determined as 
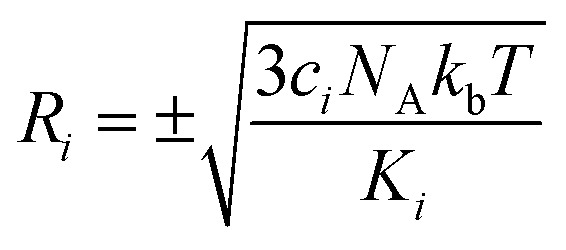
 with *N*_A_ and *k*_b_ being the Avogadro number and the Boltzmann constant, respectively. This displacement is obtained by scaling an energy with *c*_*i*_
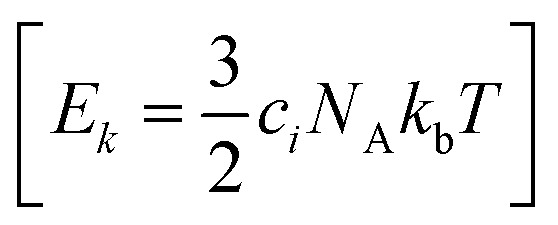
 and setting it equal to a harmonic potential 
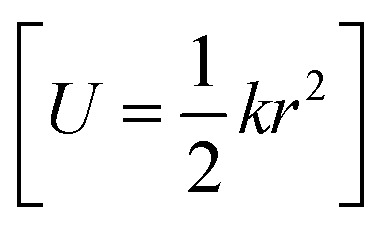
. (v) Determine the sign of the displacement *R*_*i*_ randomly using a Bernoulli distribution to sample the attractive and repulsive parts of the potential (vi) the normalized normal mode coordinates *q*_*i*_ are scaled using *R*_*i*_ giving a new set of coordinates.

Unlike the consecutive snapshots of an AIMD, NMS yields uncorrelated molecular configurations in a very efficient manner. Nonetheless, the sampling is based on a harmonic approximation of the potential well and usually only geometries close to the respective equilibrium structures are obtained. For larger displacements and large amplitude motions, the harmonic approximation breaks down. Thus, NMS is often used in conjunction with alternative sampling strategies or followed by adaptive sampling.^3^

#### 4.1.3 Diffusion Monte Carlo

Diffusion Monte Carlo (DMC) can be used to determine the zero-point energy (ZPE) and wavefunction of a molecule by appropriately, yet randomly, sampling the configurational space.^155^ The foundation of DMC is the similarity of the imaginary time SE18
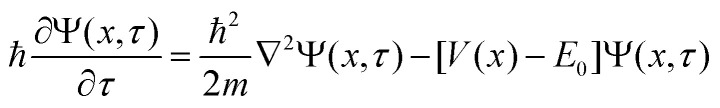
with the diffusion equation with a sink term allowing random-walk simulations to estimate the ZPE and wavefunction.^156^ Given a molecule, a set of walkers is initialized (usually at some energy minimum), propagated randomly at each time step *τ* and used to represent the nuclear wavefunction. In one dimension, the displacement assigned to each of the walkers is given by^156^19
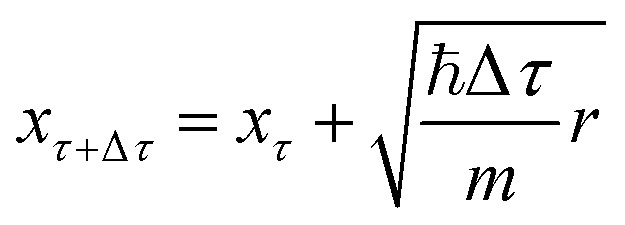
where *x*_*τ*_ corresponds to coordinates at time step *τ*, Δ*τ* is the time step of the random-walk simulation, *m* corresponds to an atomic mass and *r* is a random number drawn from a Gaussian distribution, 
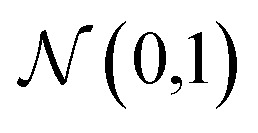
. Once the walkers are randomly displaced following eqn (19), their potential energy *E*_*i*_ is determined. Based on *E*_*i*_ with respect to a reference energy *E*_*r*_, a walker might stay alive, give birth to a new walker or can be killed following the probabilities below:

20 *P*_death_ = 1 − e^−(*E*_*i*_−*E*_*r*_)Δ*τ*^ (*E*_*i*_ > *E*_*r*_)

21 *P*_birth_ = e^−(*E*_*i*_−*E*_*r*_)Δ*τ*^ − 1 (*E*_*i*_ < *E*_*r*_)

Once the probabilities have been determined, the dead walkers have been eliminated and new walkers are initialized, *E*_*r*_ is adjusted following22
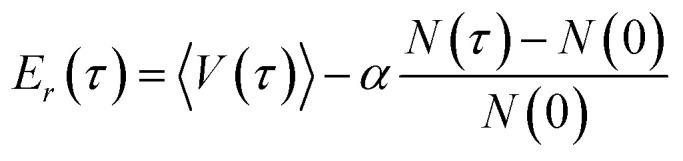


The averaged potential energy of the alive walkers is given by 〈*V*(*τ*)〉, *α* governs the fluctuation in the number of walkers and is a parameter, and *N*(*τ*) and *N*(0) are the number of alive walkers at time step *τ* and 0, respectively. The ZPE is then approximated as the average of *E*_*r*_ over all imaginary time.^155,156^

The geometries sampled using the DMC scheme are physically meaningful (the ensemble of walkers represents the nuclear ground state wavefunction) and efficiently obtained by only using energies. In comparison to AIMD, the DMC scheme has the advantage that it samples configurations up to the ZPE, which becomes larger for bigger molecules. The (quantum) exploration of a PES using DMC is typically done after a first PES has been fitted and is used to refine the reference data set.^157^ DMC has been proposed as a tool to detect holes (regions on a PES that have large negative energies with respect to the global minimum) in ML based PESs.^157^ These holes are caused by insufficient data in specific regions in configuration space, for which a NNP without any underlying physical knowledge leads to artifacts. As an adaptation, DMC with artificially reduced masses has been proposed to locate holes more efficiently due to the larger random displacements (which are proportional to 
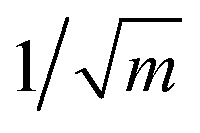
, see eqn (19)).

### 4.2 Validation and refinement of the data set

These holes were found to exhibit energies with large negative values.^158^ After an initial PES is fitted, a thorough evaluation of the PES to discover any holes is needed. For this reason, the family of active learning schemes which comprise algorithms to systematically generate reference data sets have gained considerable attention.^159^ The necessity for more elaborate sampling schemes is related to the impracticality of an exhaustive sampling of a PES and the high computational cost of extensive *ab initio* calculations. Typically, a first PES is trained on reference data based on representative configurations. This is followed by suitably extending the data set in an iterative fashion in which similar configurations are avoided and configurations from underrepresented regions of the PES are found and included into the data set.^159^ This approach is usually termed adaptive sampling (or on-the-fly ML).^160,161^ Therefore, a requirement for ML models to autonomously select new reference data is the availability of an uncertainty estimation. If a defined uncertainty threshold is exceeded for a particular configuration electronic structure calculations are performed and used to extend the reference data.

#### 4.2.1 Uncertainty estimation

Given the breadth of NN methods (or ML methods in general), various approaches for uncertainty estimation exist. One of the most popular methods is query-by-committee.^159^ This approach involves training/fitting a number of individual NNPs (*e.g.* starting from different parameter initialization or on different splits of the reference data set) and using the ensemble for predictions. In regions of the configuration space where sufficient data is available the predictions of the different models agree well. Conversely, the predictions for configurations for scarcely sampled regions will diverge rapidly, and can be used to autonomously select new configurations. A possible uncertainty metric for NNPs is^152^23
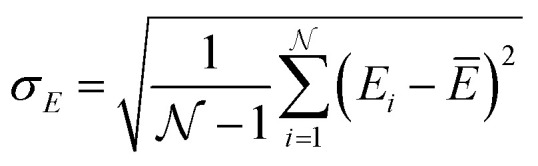
with 
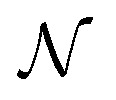
 being the number of individual models, *E*_*i*_ an individual energy prediction and the average of all energy predictions, *Ē*. Similar metrics can certainly also be adapted to other properties including the forces acting on the atoms *α*:^152^24
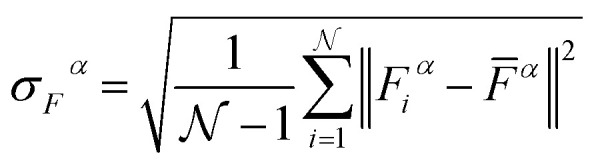


The use of query-by-committee requires the training of several independent models which incurs a high computational cost to obtain the uncertainty. In addition to this, it has been found that the uncertainty estimated by NNP ensembles are often overconfident.^162^ As a solution to this bottleneck, methods that obtain the uncertainty in a single evaluation have been proposed. Some us^76^ recently introduced a modification of the PhysNet architecture that allows the calculation of the uncertainty on the prediction through a method called deep evidential regression.^163^ Using this method, the energy distribution of the system is represented with a Gaussian and its uncertainty as a gamma distribution. With this approach, it is possible to obtain the prediction and the uncertainty of the prediction in one single calculation. Other possibilities for the prediction of uncertainties include the use of Bayesian NNs, however, they imply a larger computational cost than the previously described methods.

#### 4.2.2 Elaborate sampling techniques

With the availability of an uncertainty measure and an initial PES, geometries from underrepresented regions on the PESs can easily be identified: the initial PES is used to guide the sampling of new structures (by MD, DMC, metadynamics, …) and if the uncertainty measure (*e.g. σ*_E_) exceeds a threshold, *ab initio* calculations are performed for the geometry and the data set is suitably extended. These more systematic approaches of generating reference data sets offer a number of advantages over random methods. Since including similar configurations is avoided and new data is only added for scarcely sampled regions, the approaches are clearly more data efficient requiring less expensive quantum chemical computations. Additionally, since the NNP that is used to guide the sampling of new geometries is topologically very similar to the *ab initio* PES it is assured that configurations, that are similar to the configurations visited in AIMDs, are sampled. The quality of the uncertainty estimate is crucial for all adaptive sampling schemes. While an over-confident estimate leads to an inaccurate PES (in the worst case holes are overlooked) an under-confident estimate leads to the inclusion of redundant configuration and unnecessary, computationally expensive *ab initio* calculations. Zipoli and coworkers report that adding new configurations based on uncertainty estimation from an ensemble of NNPs does not show significant differences from random sampling.^162^ Contrary to that, Pernot^164^ and Zheng *et al.*^165^ find that querying the uncertainties from ensembles are well suited for outlier detection and adaptive sampling. This clearly indicates the necessity for future studies exploring more elaborate sampling techniques.

## 5 Knowledge transfer

Most ML algorithms (foremost deep learning) heavily rely on abundant training data to extract the underlying patterns in very complex data. This severe data dependence is one of the major drawbacks to deep learning.^166^ The collection of big data sets is a cumbersome and expensive task impeding the generation of large, high-quality data sets. While this time-consuming endeavor might be possible for some areas of application (*e.g.* manually labeling images for an image recognition task) insufficient training data/data scarcity is an inevitable problem in other domains (*e.g.* drug discovery).^166,167^ Thus, transfer learning (TL)^166,168^ and related approaches including Δ-ML,^169,170^ dual-level Shepard interpolation,^171^ multifidelity learning^172^ or the multilevel grid combination technique^173^ have been proposed to circumvent the severe data dependence/scarcity or expensive labeling efforts by knowledge transfer. Thereby, exploiting the knowledge acquired by solving one task (a source task) to solve a new, related task (a target task) forms its common ground.^168^

Besides addressing the data scarcity dilemma, knowledge transfer also helps reducing training times, computer resources (which both are significant for large data sets/models^174^) and their energy consumption. Recently, the CO_2_ emission for training common natural language processing (NLP) models has been studied, which, depending on their size, can exceed a car's lifetime CO_2_ emission.^175^

**Fig. 5:**
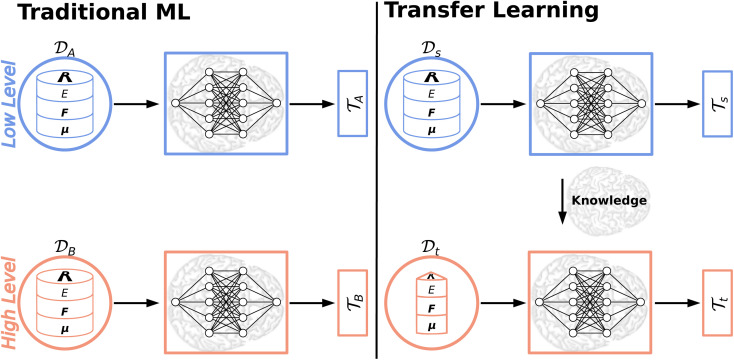
Illustration of the difference between traditional ML and TL approaches. In traditional ML, two different models are trained for two different tasks 
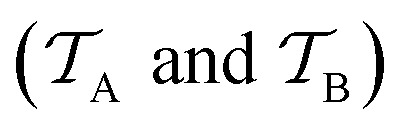
, although the two tasks might be related (*e.g.* predicting the MP2 and the CCSD(T) energy of a given configuration). In TL, however, the knowledge gained from solving a source task 
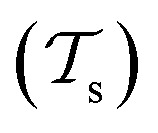
 in the source domain 
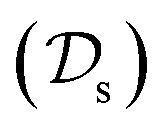
 is used to solve a target task 
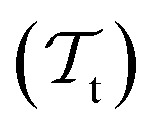
 (*e.g.* by fine-tuning the weights and biases). In the context of PES generation, typically a (global) PES is developed at a low level of theory and then transfer leaned with less data calculated at a considerable higher level of theory (*e.g.* CCSD(T)).

Traditional ML problems usually proceed in a domain 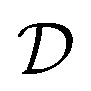 and try to solve a specific task 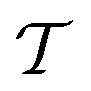. In the context of molecular PESs, the domain 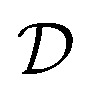 is a set of molecular configurations (defined by {**R**,**Z**}) with their associated descriptors (see Section 3.1) and the task involves the prediction of the corresponding energies *E*_*λ*_^BO^(**R**) (eqn (3)). Considering two domains (a source 
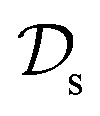
 and a target domain 
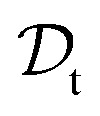
) and two learning tasks (
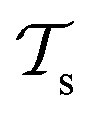
 and 
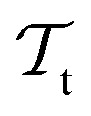
) from the perspective of traditional ML, two separate machines are trained to solve the two tasks (see Fig. 5). In contrast, TL circumvents learning to solve both tasks from scratch by facilitating the learning of 
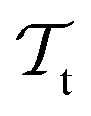
 with knowledge from 
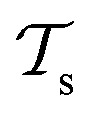
 (see Fig. 5). Here, the domains and/or tasks can differ for TL giving rise to three distinct cases.^167,168^ (i) The domains are the same, 
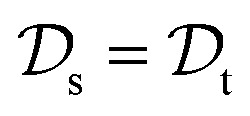
, while the tasks differ, 
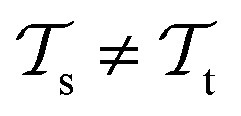
. This situation can, *e.g.*, be found for TL between molecular properties (inductive learning) (ii) the domains differ, 
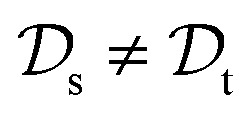
, while the tasks remain the same 
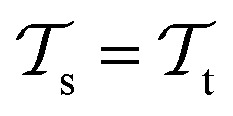
. This corresponds to transductive learning and can be found for TL between different molecular data sets. (iii) Both, the domains and the tasks differ, 
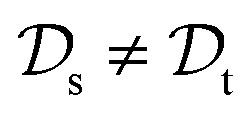
 and 
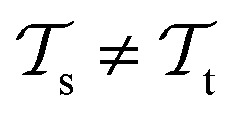
. All three subsettings have in common that they try to learn/improve the target predictive function *f*_t_(·) of 
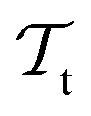
 in 
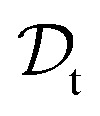
 using the knowledge in 
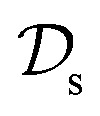
 and 
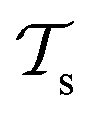
 which is the definition of TL.^168^

The training of NNPs typically requires thousands to tens of thousands of *ab initio* calculations even for moderately sized molecules, which often limits the quantum chemical calculations to the level of density functional theory (DFT). If highly accurate molecular properties are needed, researchers usually resort to the coupled cluster with perturbative triples (CCSD(T)) level of theory. This “gold standard” – CCSD(T) – scales as *N*^7^ (with *N* being the number of basis functions),^176^ which makes calculating energies and forces for large data sets and larger molecules impractical. Thus, TL^50,177–180^ and related Δ-learning approaches^170,181–183^ gained a lot of attention in recent years and were shown to be data and cost effective alternatives to the “brute force” approach in quantum chemistry: a low level PES based on a large data set of cheap reference data (*e.g.* DFT) is generated first, which then is used to obtain a high level PES based on few, well chosen high level of theory (*e.g.* CCSD(T)) data points.

### 5.1 Deep transfer learning

Deep TL^167^ combines deep NN architectures with TL among which fine-tuning is the most commonly used technique. Fine-tuning, which is a parameter-based TL technique, assumes that the weights and biases of a deep NN that was trained on a source task 
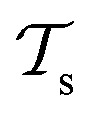
 contain useful information to solve a (related) target task 
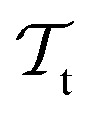
. In the context of molecular PESs, a lower level (LL) PES is obtained by training a deep NN on a large data set of energies/gradients determined at a low level of theory. Then, the parameters (weights and biases) of the LL PES are migrated to the target model for which they serve as the initialization (a good initial guess). The target model (*i.e.* the transfer learned model) is then fine-tuned (retrained) on a small data set of high-level of theory energies/gradients. The fine-tuning technique that migrates the parameters of a LL PES to a high level (HL) PES is shown in Fig. 5.

There are certain subtleties when applying TL in practice. TL can be performed without any further restriction to the fine-tuning for which all weights and biases are allowed to adapt to the new HL data. Conversely, it is possible to fix the weights and biases of particular layers. Usually, the first hidden layers are fixed and only the last layer(s) are allowed to adjust (alternatively a new, final layer can be added keeping the LL model as is). Fixing a portion of the NN parameters limits its flexibility but might help in reducing overfitting for small data sets. Recently, TL in combination with NNs was used for structure-based virtual screenings of proteins.^184^ The authors found that fine-tuning a full NN worked best for kinases, proteases and nuclear proteins, however, fine-tuning only the final layer yielded better results for G-protein-coupled receptors (GPCRs). They speculate that this is caused by the limited and less diverse data for GPCR targets. Besides the need to avoid overfitting, it is imaginable that for NNs that employ learnable descriptors of the atomic/molecular configuration it might be beneficial to freeze the parameters that are used to learn the descriptor for the fine-tuning step. Instead of freezing a portion of the layers, fine-tuning with differential learning rates^185^ (*i.e.* having different learning rates for different parts of the NN) could allow minimal changes to early layers (*e.g.* where the descriptors are learned) and larger adjustments to the later layers. Although empirical rules are followed in the community, accepted criteria for choosing TL methods are essentially nonexistent.^167^

### 5.2 Δ-Machine learning

The Δ-machine learning approach was developed in the context of kernel-based methods and is motivated by the fact that the heaviest burden in quantum chemical calculations is the determination of a tiny energy contribution to a (approximate) total energy.^170^ The approximate energy often is able to describe the general chemistry/physics of a given system, while the determination of the “Δ” comes at a tremendous computational cost due to adverse scaling with system size of correlated electronic structure methods. For a molecular property, the Δ-ML prediction is modeled as a LL value plus a correction towards a HL value following25
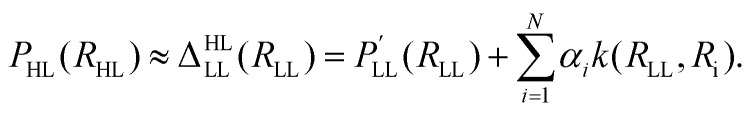


The high level property *P*_HL_ (*e.g.* enthalpy *H*_HL_) at a relaxed molecular geometry (*R*_HL_) is approximated as a related property 
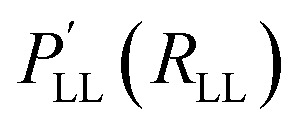
 (*e.g.* energy *E*_LL_) obtained at the LL plus a correction term^170^ that is obtained from ML (reference 170 employed Slater type basis functions *k* and kernel ridge regression (KRR) to obtain the regression coefficients *α*_*i*_). The Δ-ML approach as defined in eqn (25) allows modeling changes in level of theory (*e.g.* DFT → CCSD(T)), molecular property (*e.g.* energy → enthalpy) and molecular geometry. Although the Δ-ML approach is often used in conjunction with kernel-based methods, a correction PES Δ (*i.e. V*_HL_ = *V*_LL_ + Δ) can also be learned using NNs.^186^ The resulting HL PES *V*_HL_ can either be used directly (requiring the evaluation of two models) or can be used as a proxy to generate a larger data set for a final training containing many, though approximate, HL points.^186^ As is common for the ML field, different flavours of Δ-ML exist.^146,170,172,173,181,182,186–189^

Recent work proposed “Δ-DFT” that uses Kohn–Sham (KS) electron densities *ρ*^KS^ to correct the DFT energy towards, *e.g.*, a coupled cluster energy following

26 *E*^cc^ = *E*^DFT^[*ρ*^KS^] + Δ*E*[*ρ*^KS^]

using KRR.^146^ While the formalism of DFT and wavefunction based approaches (such as CCSD(T)) differ radically (also note that the CCSD(T) density is not routinely calculated and not needed to obtain the CCSD(T) energy), the “learnability” of DFT and CCSD(T) energies from KS densities was studied alongside the Δ − DFT approach. The authors find starting from *ρ*^KS^ learning DFT and CCSD(T) energies directly is associated with approximately the same effort. However, learning Δ*E*[*ρ*^KS^] was more efficient and yielded lower out-of-sample errors at smaller training set sizes.^146^

## 6 Exemplary applications of NNPs in molecular simulations

The high flexibility of NNs allows the representation of PESs for a wide range of chemical systems and reactions as long as a sufficiently large reference data set is available from *ab initio* computations at a sufficient level of theory to correctly describe the physics in the system. This section presents several typical applications of NNPs in molecular simulations.

### 6.1 Gas phase spectroscopy

In a recent review, Manzhos and Carrington report advances of NNPs and applications in classical and quantum dynamics of small and reactive systems.^125^ They point out that for small systems modern NNPs are still outperformed by permutationally invariant polynomial (PIP^33,36^) methods in terms of PES fitting error which, however, does not translate to significant deviations in computed observables such as vibrational frequencies.^190^ As an example, the RMSE of a Gaussian process regression (GPR) model potential (0.017 kcal mol^−1^, 5.98 cm^−1^) is half of that of a NNP (0.034 kcal mol^−1^, 12.03 cm^−1^) with regard to 120 000 reference points for formaldehyde. However, the RMSE of the first 50 (100) predicted vibrational frequency levels with respect to their reference is 0.43 cm^−1^ (0.82 cm^−1^) for the NN and 0.46 cm^−1^ (0.82 cm^−1^) for the GPR potential. When the potential models are fitted to a subset of reference points with high significance for the vibrational frequency prediction, the RMSE of the first 50 (100) predicted vibrational frequency levels differs substantially with 0.21 cm^−1^ (0.30 cm^−1^) for the NN and only 0.04 cm^−1^ (0.06 cm^−1^) for the GPR model.^125,191^

**Fig. 6:**
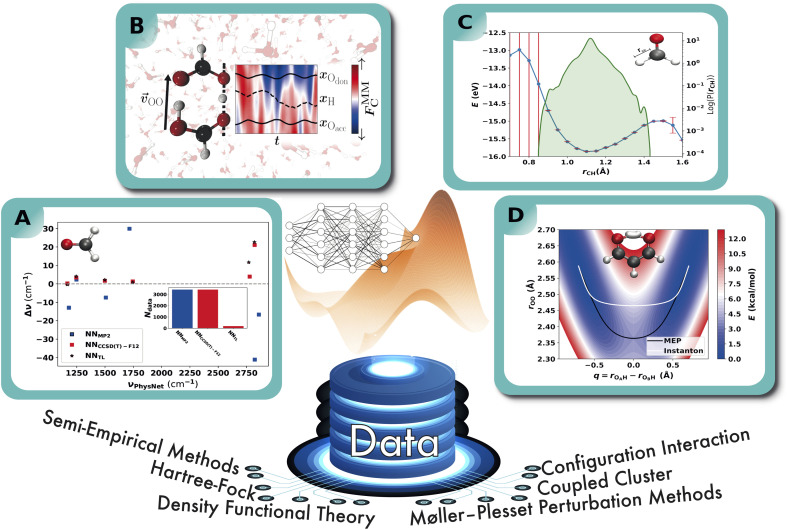
Schematic representation of the exemplary applications of NNPs. A: performance of a NNPs based on MP2/aVTZ and CCSD(T)-F12/aVTZ-F12 with respect to experiment. NNPs trained from scratch are compared to the more-data efficient TL approach and the anharmonic frequencies are obtained from VPT2 calculations.^179^ B: double proton transfer in formic acid dimer from mixed ML/MM/MD simulations.^192^ The time series next to the molecular structure shows the variation in the background solvent field depending on time across one proton transfer event. C: 1D cut of the PES of the C–H bond in formaldehyde (upper right) calculated with the PhysNet evidential model (blue curve). Red bars indicate the predicted variance by the model. The green distribution shows the logarithm of the probability distribution of the distances covered by the training set. D: the two-dimensional projection of a NN-trained PES of CCSD(T) quality for proton transfer in malonaldehyde. The white and black traces are the instanton and minimum energy paths, and the PES is used to calculate tunneling splittings.^193^

The application of NNPs to determine anharmonic vibrational frequencies in combination with TL has been studied in ref. 179. For that purpose, a NN of the PhysNet type is trained on *ab initio* energies, forces and dipole moments and employed in second order vibrational perturbation theory (VPT2) calculations that are directly compared to their experimental counterpart. A total of eight molecules are studied from which the results for formaldehyde are shown in Fig. 6A as it allows a good comparison of a TL scheme with a model that is trained “from scratch” due to its small size. A PhysNet model that is trained on MP2 data (NN_MP2_) yields errors up to 40 cm^−1^ with respect to the experimental values, while the CCSD(T)-F12 model (NN_CCSD(T)-F12_) has a maximum deviation of ∼20 cm^−1^. Both NN_MP2_ and NN_CCSD(T)-F12_ were trained on roughly 3400 *ab initio* energies, forces and dipole moments, for which the computation at the CCSD(T)-F12 level of theory requires high computational effort. In contrast, 6% of the CCSD(T)-F12 reference points are sufficient to transfer learn a NN_MP2_ model and achieve an accuracy that is within ∼7 cm^−1^ of NN_CCSD(T)-F12_ trained on the full reference set from scratch.

### 6.2 Condensed phase simulations

Even though NNPs scale more favourably with the number of atoms, the construction of a reference data set for molecular compounds still requires several thousand *ab initio* calculations. As NNPs are mathematical representations of the input data and are uninformed about the underlying physics governing intermolecular interactions, their extrapolation capabilities are rather limited. This also concerns the transferability of NNPs optimized on smaller molecular clusters towards larger clusters or even periodic systems. This issue has been addressed recently, for instance, by Kästner and coworkers on liquid water and Marx and coworkers on protonated water clusters using NNPs.^194,195^

Kästner and coworkers train a Gaussian moment NN (GM-NN) model on DFT rev-PBE-D3 reference data of water cluster configurations produced by *ab initio* MD simulation at 150, 300 and 800 K, and study its transferability to a periodic bulk water system with 64 molecules from *ab initio* MD simulation at 400 K.^194,196^ The GM-NN model trained on clusters containing 30 to 126 water molecules can reproduce the total energy of the periodic bulk water system well, although with a slightly broader error distribution as for the model trained on the periodic system. The potential energy predicted by the cluster model for the periodic systems are also arbitrarily shifted mainly due to the differences in the non-periodic and periodic computational system setup. MD simulation of a periodic water box at 300 K with the model potentials trained on clusters (cluster model) and periodic reference data (bulk model) produce radial distribution function that agree well and X-ray diffraction spectra are close to experimental ones. The computed water molecule self-diffusion coefficients and equilibrium density from simulations with the cluster model are about 18% larger (2.15·10^−9^ m^2^ s^−1^ and 1.02 g cm^−3^) than with the bulk model (1.82·10^−9^ m^2^ s^−1^ and 0.86 g cm^−3^) but closer to the respective experimental values (2.41·10^−9^ m^2^ s^−1^ and 1.00 g cm^−3^). Detached from the evaluation of the rev-PBE-D3 method and MD setup to accurately reproduce experimental water properties, the case study shows transferability of the cluster model to reproduce bulk properties. However, the authors mention that further studies are necessary to get insights into the deviation in the computed properties of both models as both water cluster and periodic water system are based on the same physical–mathematical description. Only water molecules closer to the cluster surface experience different strain energy than bulk water due to the lack of bonding partners.

Great transferability is also shown by Marx and coworkers using a HDNNP model trained on protonated water cluster H^+^(H_2_O)_*n*_ (*n* = 1–4) with up to four water molecules to representing the PES of a protonated water hexamer H^+^(H_2_O)_6_.^73,195^ The reference data for the protonated water clusters *n* = 1–4 were produced by an automatic fitting procedure that performs DFT based *ab initio* MD and path integral MD (PIMD) simulation at 1.67, 100 and 300 K to sample relevant configurations. Within a repeated fitting procedure, holes in the reference data set are detected by estimating the uncertainty as described in section 4.2.1 or configurations were included where the local descriptors (ACSFs) of configurations in the MD simulation leave the range of the reference data set.^197^ A final data set is created from reference data of the configurations computed at CCSD(T*)-F12a/aug-cc-pVTZ level of theory. Extrapolation of the NN model trained on the smaller cluster *n* = 1–4 to configuration of the protonated water hexamer yields a mean absolute energy error about three times higher than for the original training data set that is 0.026, 0.031, 0.038 kcal mol^−1^ (0.11, 0.13, 0.16 kJ mol^−1^) per atom against 0.007, 0.010, 0.012 kcal mol^−1^ (0.03. 0.04, 0.05 kJ mol^−1^) per atom from the sampling procedure at 1.67, 100 and 300 K, respectively.^195^ Again, an arbitrary shift is added to the predicted energies of the hexamer to minimize the error between the predicted and the reference energies. The ability to extrapolate is illustrated by comparing the potential energy sequence for 25 fs between an *ab initio* MD and the MD simulation using the NNP. It is further noticeable, that the extrapolation towards the hexamer potential failed in PIMD simulations for which unphysical configurations are reached if the NNP is trained only on tetramer configurations (*n* = 4). The authors conclude that the transferability towards larger cluster sizes improves if smaller clusters are included within the training data set.

### 6.3 Reaction rates

The reaction of methane with molecular oxygen is one of the most fundamental but highly complex combustion processes involving more than one hundred different reaction steps as shown by experiments.^198^ Zhu and Zhang report MD results of the combustion reaction including 100 methane and 200 oxygen molecules at 3000 K simulated for 1 ns.^199^ They used the DeepMD model potential that was fitted to reproduce 578731 reference DFT energies at the MN15 level of theory.^115,200^ In their simulation they detected 505 molecular species and 798 different reactions where 130 reaction steps are also reported from experiments.^198^ A selection of computed reaction rates deviates from experiment by up to two orders of magnitude, but combustion reactions usually involve the formation of radical species, that might require a non-adiabatic molecular dynamics approach which are highly non-trivial.

Marquetand and coworkers applied the SchNarc approach to investigate the photodissociation reaction of tyrosine that shows a dissociation channel of a hydrogen radical with a chemically non-intuitive path which is called roaming.^201^ Roaming was originally explored experimentally and computationally in formaldehyde by Bowman and coworkers in 2004 but real-time experimental observation were not achieved until 2020.^202,203^ The NNP is learned to reproduce 29 energy values and force values for electronic singlet and triplet states and 812 spin–orbit couplings. They simulated over 1000 trajectories of at least one picosecond which, in comparison, would take over eight years for *ab initio* MD simulation on a high-performance computer. About 17% of the trajectories show the roaming of the hydrogen atom in photoexcited tyrosine that lead to a higher ratio of subsequent further fragmentation than in non-roaming trajectories. This application marks a major step forward towards atomistic simulations of photoexcitation reactions in larger molecules like proteins that lead to further insight in, *e.g.*, photosynthesis, harmful photodegradation or drug designing for phototherapy.

### 6.4 Hybrid ML/MM simulations of solvated systems

The use of NNPs as force fields promotes the performance of MD simulations in comparison to the *ab initio* MD counterpart. But even if the computational cost of NNPs scales by a similar factor of ∼O(*N*^1–2^) as empirical force fields do, due to their more compact and explicit functional form empirical force fields are considerably more efficient in general. Thus, a significant speed-up in MD simulations can be achieved by decomposing the force field into a contribution from a NNP (ML part) for, *e.g.*, a solute of interests or a reactive center in a protein, an empirical force field (MM part) for solvent molecules or protein backbone structures, and a coupling (or embedding) between the ML and MM parts. This approach is well known and applied in QM/MM MD simulations.^204^

One straightforward approach was pursued to investigate the double proton transfer reaction in cyclic formic acid dimers and the electrostatic impact of a water solvent on the reaction rate as shown in Fig. 6B.^192^ Here, a PhysNet model was trained with a reference data set including formic acid dimers and monomers in the gas phase at MP2/aug-cc-pVTZ level of theory. The model accurately reproduces the energies, forces and molecular dipole by assigning atom centered charges.^51^ The interaction potential between formic acid and the TIP3P water solvent consists of Lennard-Jones terms with parameters from the CGenFF^205^ force field and electrostatic interactions between the atom charges from the TIP3P^206^ water atoms and the configurational dependent PhysNet charges of the formic acid atoms. The advantage is the lower computational cost to produce trajectories with lengths of multiple nanoseconds to statistically sample the raw double proton transfer events with a rate of just 1 ns^−1^ at 350 K. Furthermore, the NNP fit inherently includes the coupling of the reactive potential path of the proton transfer with other structural dependencies such as the C–O bond order of the acceptor and donor oxygen and the dimer dissociation reaction into formic acid monomers. On the other hand, such an approach does not include the mutual polarization of the formic acid charges and the water solvent which, in the present case, is however expected to be small. This is akin to a mechanical embedding known from QM/MM schemes.^207^

Applications of electrostatic embedding in ML/MM simulation are reported by Riniker and coworkers as well as Gastegger and coworkers.^208,209^ Here, the ML-MM interaction potential includes the polarization of the ML system by the electric field originating from the MM compounds. Riniker and coworkers modified the HDNNP by providing two sets of local descriptors for just ML solute atoms and surrounding MM solvent atoms, separately. The model is trained to reproduce either the ML atom potential and the electrostatic component of the ML–MM atom interaction itself (pure ML/MM) or in accordance of the Δ-learning approach an energy correction of both components to improve from computational cheap tight-binding DFT result towards more accurate reference data ((QM)ML/MM).^111,208^ This approach demands larger reference data sets from QM calculations to sample solute configurations with different solvent distribution where the solvent is represented as their respective MM point charges. However, the Δ-learning (QM)ML/MM approach applied to tight-binding DFT computations have been shown to achieve higher accuracy even with fewer reference samples than the pure ML/MM model.

The accuracy is illustrated by running *NPT* simulations of *S*-adenosylmethionate and retinoic acid in explicit water solvent at 298 K and 1 bar using the pure ML/MM and the (QM)ML/MM model for 5000 and 2000 integration steps of 0.5 fs, respectively, and comparing it to reference QM/MM results.^208^ The mean absolute error for the (QM)ML/MM model is up to one magnitude lower with 1.4 kcal mol^−1^ (5.8 kJ mol^−1^) and 12.6 kcal mol^−1^ (52.8 kJ mol^−1^) than the pure ML/MM model with 4.3 kcal mol^−1^ (18.1 kJ mol^−1^) and 17.9 kcal mol^−1^ (74.9 kJ mol^−1^). One integration step with the (QM)ML/MM model takes less than a second on 1 CPU while the reference QM/MM model at DFT BP86/def2-TZVP level is about 3 magnitudes slower with about 60 to 80 minutes on 4 CPUs. A potential disadvantage of the (QM)ML/MM model is that certain solute configurations at the tight-binding DFT level may fail to converge or converge only slowly, *e.g.*, during a reaction.

Gastegger and coworkers presented the FieldSchNet model, a modification of the SchNet model that includes energy contributions from interactions between predicted atomic charges and dipoles, but also with an external field such as the electric field originating from a set of point charges.^85,209^ The advantage of such elaborated models is the sensitivity of the potential energy to changes in atomic positions, electric and magnetic fields that enable the computation of response properties such as forces, molecular dipole moments, polarizabilities, and atomic shielding tensors that are crucial for the direct prediction of, *e.g.*, IR, Raman and NMR spectra. As the atomic charges and dipoles of the ML treated system respond to the external field caused by MM atoms point charges, this model is considered to be electrostatic embedding. Consequently, it has the same requirement for additional sampling of ML system configurations in different arrangements of MM atomic point charges as the model of Riniker and coworkers described above.^208^

For ethanol in vacuum, PIMD simulations with FieldSchNet yield excellent agreement in terms of frequency shifts and widths between predicted IR/Raman spectra and experimentally measured ones. For liquid ethanol, IR spectra were predicted from MD trajectories with an explicit ML/MM solvent model of one ML treated ethanol molecule in a MM treated ethanol solvent. The explicit ML/MM approach shows great agreement with experimental IR spectra in the low frequency region and a blue shift for the C–H and O–H stretch vibrations bands in the high frequency range due to missing anharmonicity effects by the MD approach. MD simulations with an implicit PCM solvent model do not yield an IR spectra with significant differences from gas phase spectra as it fails to capture hydrogen bridging between ethanol molecules.^210^ However, the applied ML/MM model still predicts the intermolecular ML–MM potential between ML ethanol and the MM solvent by the CGenFF^205^ force field with fixed atomic charges. The implementation of the electrostatic interaction between predicted atomic charges and dipoles by FieldSchNet and the MM point charges is a highly non-trivial task and would further increase the computational costs. It limits the application range to systems where the ML–MM interaction potential is sufficiently well described by the MM force field that may not work for dynamics with complex configurational changes or chemical reactions.

Electrostatic embedding in the QM/MM approach (and the ML/MM approach)^208^ includes the QM-MM electrostatic interaction and the polarization of the QM system by the electric field of the MM atoms but not *vice versa*. The highly expensive task to approximate the polarization of the MM system by the electric field of the QM system is part of polarizable embedding schemes.^211^ An analogue for the hybrid ML/MM model is developed Westermayr, Oostenbrink and coworkers with their buffer region NN approach (BuRNN).^212^ Here, a buffer region around the ML atoms is defined by a cutoff sphere to select MM atoms within the sphere. The ML and selected MM atoms are the input to a modified SchNet model to predict the potential energy between the ML atoms, the ML-MM interaction energy and a polarization correction energy to the classical MM potential of the MM atoms within the buffer sphere to match reference potential data. The modified SchNet model also predicts atomic point charges for the ML atoms and MM atoms within the buffer region, which are used to compute the electrostatic interaction to the remaining MM atoms in the system outside the buffer region. The potential energy of the atoms in the inner region are predicted by a modified SchNet model. As for electrostatic embedding, potential energy and charge distribution of the ML system are impacted by the MM atoms within a buffer region and, additionally, interaction energy and atomic charges of the respective MM atoms are impacted by the ML system. A major disadvantage is the high computation cost for the reference data set, that requires two quantum electronic calculation for configuration samples of (1) the ML system and MM atoms in the buffer region and (2) the MM atoms in the buffer region alone to predict the polarization correction term.

The BuRNN approach was applied to a hexa-aqua iron(iii) complex simulated by a ML treated Fe^3+^ ion in a water solvent described by the SPC model. A buffer region was defined by a cutoff radius of 5 Å around the Fe^3+^. MD simulation of 10 ns shows smooth diffusion of water molecules entering and leaving the buffer region and reveal power spectra that match the low frequency bands around 180, 310 and 500 cm^−1^ observed in experiments very well. Radial and improper and distributions between Fe^3+^ and the oxygens of the coordinated water match with distributions from QM/MM simulation with electrostatic embedding and are within experimental estimations.

All the presented applications show an active field of developments in hybrid ML/MM approaches towards accurate MD simulation of solutes or reactive species in the presence of a solvent. A major gain in computational efficiency and much longer simulation times at comparable accuracy are achieved by replacing QM methods with a NNP. However, the effort to generate a reference data set that sufficiently samples the relevant configurational space of the ML system in combination with different solvent configuration depends significantly on the embedding scheme. The simplest mechanical embedding scheme only requires a converged NNP that predicts the total energy, forces and the charges of the ML system in the gas phase but it neglects polarization of the MM atoms.^192^ In comparison, NNPs based on electrostatic embedding require additional sampling with MM atom configurations included as point charges. MD simulation using ML/MM approaches with electrostatic embedding show great agreement with MD simulation of respective QM/MM simulation at the same level of theory as the reference data set.^208^ The increase in the quality to describe the impact of the MM solvent on the properties of the ML system is also demonstrated by accurate computational reproduction of experimental IR and Raman spectra.^209^ The most complex polarization embedding scheme allows the most complete description of the ML system with the MM environment, but requires more costly reference computations.^212^ Even a QM/MM model using polarization embedding is significantly more challenging in terms of computational effort and implementation than the electrostatic embedding schemes.^211^

## 7 Applications based on but beyond PESs

Up to this point PESs were used in explicit simulations to determine experimental observables from dynamics or Monte Carlo simulations. However, quantum nuclear dynamics or a statistically significant number of (quasi) classical MD simulations and their analysis is often a computationally demanding endeavor in itself. It would be desirable to determine, predict or estimate observables from only a limited amount of such explicit simulations and devise rapidly-to-evaluate models that predict with confidence outcomes for arbitrary input. To set the stage, the full characterization of all state-to-state cross sections for reactive triatomic systems A + BC → AB + C is considered. This problem involves ∼10^8^ transitions. Using QCT simulations, convergence of each of the cross sections requires ∼10^5^ independent trajectories to be run. Hence, for one collision energy ∼10^13^ QCT simulations would be required for a full characterization of a reactive triatomic system. This is neither desirable nor meaningful to do. Hence, despite the availability of a full-dimensional NN-based or otherwise represented PES it would be advantageous to reduce the computational burden of explicitly sampling the PES in this case and the task is to extract as much information as possible from only a limited number of simulations.

The two problems considered further below concern the prediction of final states or final state distributions for atom + diatom reactions and predicting thermal rates for bimolecular reactions. Both problems can, in principle, be solved accurately for carefully chosen systems which provides the necessary benchmark to extend the range of applicability of the approaches described below to larger systems.

### 7.1 Final state distributions for atom + diatom reactions

Exhaustive enumeration and characterization of final state distributions from bimolecular reactions is particularly relevant in combustion and atmospheric re-entry (hypersonics). The particular interest is rooted in need to devise more coarse-grained models for the macroscopic (in space and time) modeling of the chemistry and physics of reactive flows but based on accurate microscopic information.^213,214^ For atom + diatom reactions (A + BC → AB + C) this involves complete enumeration of all state-to-state reaction probabilities. As mentioned above, this problem can - in principle – be addressed by brute-force sampling. But this is neither practical nor desirable.

For this reason, ML-based models were devised that allow to either predict final states or final state distributions from discrete initial states. From quasiclassical trajectory (QCT) simulations for the N(^4^S) + NO(^2^Π) → O(^3^P) + N_2_(X·^1^Σ^+^_g_) reaction the state-to-state cross sections *σ*_*v*,*j*→*v*′*j*′_(*E*_*t*_) as a function of the translational energy *E*_*t*_ were explicitly determined for 1232 initial ro-vibrational states (*v*,*j*) which amounted to ∼10^8^ QCT trajectories in total. This compares with an estimated 10^15^ QCT trajectories required for brute-force sampling of the problem. This information was used as input to train a NN together with features such as the internal energy, the vibrational and rotation energy of the diatoms, or the turning points of the diatoms.^215^ The resulting state-to-state (STS) model is capable of predicting the cross section for a final state given an initial collision energy, the vibrational state *v* of the diatom and its rotational quantum number *j*. More recently, the approach was extended to predict entire final state distributions from discrete initial conditions, which led to the state-to-distribution (STD) model.^216^ Finally, it is also possible to devise distribution-to-distribution (DTD) models.^217^

**Fig. 7:**
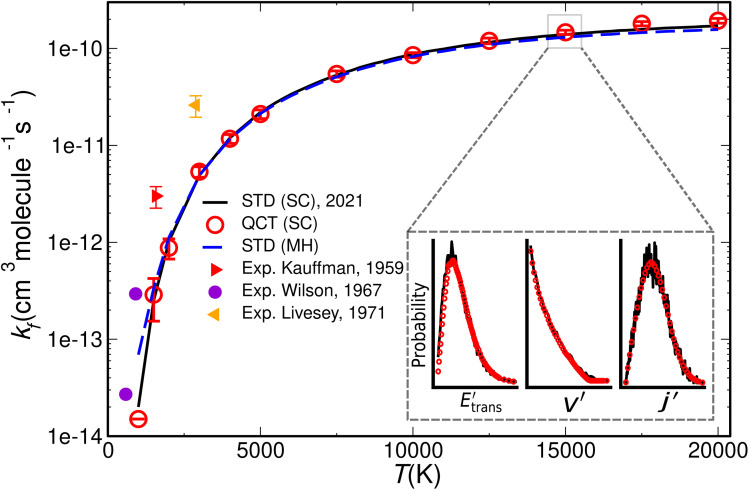
The thermal forward rate *k*_f_ calculated from QCT (open red circle) and STD model (solid black line) for the ^4^A′ state of the N(^4^S) + O_2_(X·^3^Σ^−^_g_) → NO(X^2^Π) + O(^3^P) reaction between 1000 and 20 000 K. Experimental total forward reaction rate *k*_f_ (including contributions from the doublet and the quartet states) are also shown for comparison: (red triangle),^218^ (orange triangle)^219^ and (magenta circle).^220^ A comparison is made between QCT and STD model based on model Hamiltonian *E*_trans_ (dash blue line) for the predicted distributions in the bottom right corner (inset). The evaluation is made at *T* = 15 000 K with QCT and STD evaluations marked as black and red solid lines respectively. Figure courtesy J. C. San Vicente Veliz.

The prediction quality of STS, DTD, and STD models is universally high and reaches a correlation coefficient *R*^2^ ∼ 0.98 or better between predicted and QCT-calculated reference data. From these models it is also possible to determine thermal rates as done for the N(^4^S) + O_2_(X·^3^Σ^−^_g_) reaction shown in Fig. 7 and further examples are given below. Comparison with rates directly determined from QCT simulations - which themselves are in good agreement with experiments^221–223^ – shows that the trained NNs reach accuracies better than 99% over a wide temperature range (1000 ≤ *T* ≤ 20 000) K. Thus, ML-based models based on limited input data from direct simulations on high-quality, full-dimensional PESs are a computationally efficient and accurate substitute for explicit, brute-force evaluations of the relevant properties.

### 7.2 Predicting thermal rates

Determining thermal rates is one of the major goals of computational chemistry. Carrying out such a calculation in full dimensionality, based on an accurate PES and including nuclear quantum effects is a serious computational undertaking. An accurate rate requires treating the electronic structure, representing the underlying PES, and running the (quantum) dynamics simulations at the highest possible levels and has only been done for a few selected systems. Hence, it is of great interest to develop models that can predict thermal rates based on alternative approaches.

One such effort was based on a library of ∼40 bimolecular reactions for which *T*-dependent rates from transition state theory (TST), the Eckart correction to TST, and a set of tabulated “accurate rates” from two-dimensional calculations at 8 temperatures were available.^224^ These calculations required a represented PES for carrying out the necessary dynamics simulations. The data collected was used to learn a correction to the product of the TST-rate and the Eckart correction by using Gaussian process regression. Reactions considered included the Cl + HCl H-atom exchange reaction (in 1d and 3d), the H_2_+OH → H + H_2_O and for O + CH_4_ → OH + CH_3_ which was investigated more in-depth in a separate study.^225^ The results for reactions not used in the learning procedure indicate that it is possible to obtain thermal rates close to those from explicit quantum simulations or trajectory-based quantum calculations (ring polymer MD).^226^

### 7.3 Other applications

In one recent application a mapping between local water cluster arrangement and the frequency of an embedded solute as the spectroscopic probe was used to predict water anharmonic stretch vibrations.^227^ Although this application is not dependent on and does not require a full-dimensional NN-based PES it illustrates the potential uses of a mapping between structure and spectroscopy that can be exploited in the future. Another area which links intermolecular interactions, structural dynamics and spectroscopy are ionic and eutectic liquids (ILs and ELs). A strong case for combining rigorous MD simulations with accurate, ML-based FFs for property prediction has been made for ionic liquids.^228^ ILs and ELs are characterized by strong interactions that probe the short-range part of electrostatics due to the chemical composition of the systems which consists of a high density of positively and negatively charged building blocks. For ELs a recent combination of MD simulations, two-dimensional infrared and terahertz spectroscopy was able to elucidate the microscopic structure of the liquid depending on the degree of hydration without, however, using a ML-based FF.^229^ Further improved agreement between simulations and experiments than that reported can be expected from refined intermolecular interactions.

## 8 Challenges

This section discusses several challenges the field of NN-based PESs faces. Some of the points discussed may also apply to other ML-based techniques more broadly in other branches of chemistry. As a very general opening point it is noted that one of the challenges in statistical approaches is to extract as much consolidated information, potentially including an error estimate on the prediction, from a statistical model from as little information possible. This point concerns very broadly the aspect of “data efficiency”.

### 8.1 Data management and availability

Given the tremendous computational cost and effort needed for generating robust and high quality reference data sets for PES fitting, data management and availability is a fundamental focus. Yet, the raw *ab initio* data (nuclear geometries, energies (and gradients)) of a published PES is often not publicly available, incomplete or lacks key information such as a precisely specified level of theory or the employed quantum chemical software. This could be avoided by publishing exemplary input files alongside the *ab initio* results. Some of the most popular data sets used for benchmarking NN potentials contain only equilibrium geometries and corresponding energies from different levels of theory and are used to benchmark ML methods. These include the QM7,^19^ QM7b,^230^ QM9,^231^ and ANI-1ccx^232^ databases. Databases that contain energy and gradients for equilibrium and distorted structures for different molecules include ANI-1,^233^ the refined ANI-1x^232^ and QM7-X.^234^ A popular data set that provides energies and gradients for configurations visited in MD simulations is the MD-17 dataset^235,236^ which is generated from *ab initio* MD.

On a cautionary note regarding publicly available datasets, it was reported that PESs resulting from the MD-17 data are likely to feature holes in high-energy regions which are visited for example in DMC simulations.^237,238^ Databases such as ANI-1 (ref. 233) which uses normal mode sampling for multiple species also can generate problems. Recently, it was found that redundancies in databases can compromise the prediction quality of NN models exploring chemical space.^14^ For training NNPs, the influence of the distribution of the reference points on the quality of the PES is an open question. Recent efforts in providing data sets for rigorous and global PES gave rise to the VIB5 (ref. 239) and QM-22 (ref. 238) databases that include energies (and gradients) for different molecules calculated at various levels of theory.

An often overlooked step in generating databases is the prepossessing step. It is advisable that the generated data contains as little redundancies as possible by removing correlated states to reduce the number of *ab initio* calculations and training time. Therefore, the generated database can, *e.g.*, be analyzed beforehand by unsupervised machine learning methods which have been successfully applied to evaluate MD trajectories.^240,241^ It is also important to consider that the generation of data must be application driven because the properties of interest will determine the amount of data required and should guide the selection of the sampling method. Data generation for NN-based PESs should be considered an iterative process in which it is best to start from a representative and “clean” data set that will be enriched based on the problem at hand as was recently done for tunnelling splittings in malonaldehyde.^193^

Finally, ML models are starting to face some of the same difficulties that the molecular simulation community has been dealing with.^242^ This includes the lack of standard file formats, shortage of tools for file sharing, absence of methods to ensure the quality of the generated databases, *etc.* Hence, it is worth mentioning that the young ML community has the unique opportunity to propose solutions to these obstacles before they become unbearable. In this regard, the FAIR principle^243^ (findable, accessible, interoperable and reproducible/reusable) must be taken into account. In this regard, some authors have proposed general rules for the application of ML in chemistry^244^ and in particular to PESs for small molecules.^245^ Specifically, Li and Liu^245^ proposed a checklist for reporting PESs of high-quality. As a complement to this, we propose some suggestions for providing data sets underlying molecular PESs. Data sets should:

• Provide sample input and output files for the quantum chemical software.

• Have an easy and understandable format.

• Have a consolidated structure.

• Contain raw data (at least nuclear geometries, energies (and forces)) with clearly defined units, level of theory, employed quantum chemical software.

• Have a clear description of HOW the geometries were generated.

• If possible, provide information whether the PES was developed for a particular purpose/application and whether there a known limitations.

• Be extensible.

### 8.2 Interpretability

An important ingredient for extending NN methods is the degree and confidence with which a human can understand the relationship between cause (starting database and model) and effect (result or observation, applying the model to a new task).^246,247^ This process has also been called “interpretability”, and it can be used to assess the relationships learned by the model or contained in the data used for training.^248,249^ However, for complex models like NNs the relationship between input and output is not clear as a consequence of the non-linearity and parametric complexity of the models.^56^ Therefore, it is not evident if the model is deriving the correct physics of the system from the provided data or whether it is only learning artefacts of the data which limits it's application to narrow settings in what is known as the “clever Hans” predictor.^250^ Only a few efforts have been made to derive techniques that can relate the contribution of different structural components (atom, bond type) to the predicted quantity (energy or dipole moment).^251^

Despite its importance and need, interpretability is still not a main topic in developing NNPs. A reason for this might be that the use of conventional techniques is not possible because of the continuous nature of the properties studied in chemistry.^252^ However, general guidelines have been proposed.^253^ By definition, interpretability is the missing link between the data used for training and the prediction obtained by the NNP. A better understanding of the inner processes of NNs will help to better understand the amount of data required to obtain reliable predictions, understand the completeness of the descriptor, and maybe even some new physical interactions. In contrast, the largest risk that the lack of interpretability presents is that users employ models as “black box” therefore without knowing the limitations of the model and possibly obtaining good results for the wrong reason(s).

### 8.3 Generation of robust initial models

A NNP is only as good as the data it is trained on. As a consequence, if low-quality data is used the resulting model will under-perform. This is the principle of “Garbage in-Garbage Out” which can be traced back to Charles Babbage.^254^ The NNP fitting is usually an iterative process starting from an initial reference data set. This data set ideally covers the full configurational space of the chemical system at hand with as few points as possible (note that, in principle, the number of points on a PES as well as in chemical space is ∞). While an exhaustive sampling of a PES might be possible for systems with up to 3 atoms (*e.g.* by choosing configurations on a regular grid), this becomes impossible for larger systems. Consequently, the initial sampling relies on (partly random) methods including MD or normal mode sampling (see Section 4) that all suffer from distinct weaknesses/disadvantages such as correlated structures or insufficient coverage. These weaknesses lead to additional training time, evaluations, *ab initio* calculations and ultimately to a slower and more expensive convergence of the iterative NNP fitting procedure.

Thus, the generation of data for PESs requires improved methods of (initial) sampling that can warrant sufficient coverage of the PES for a desired application with as few points as possible. An interesting prospect for the generation of PES reference data concerns spreading the data according to the “correct” distribution for different degrees of freedom resulting from methods like Boltzmann^255,256^ or Monte Carlo inversion^257^ and opens the possibility of deriving interactions from experiments.^258^ Other solutions might come from the application of information theory to ensure a number of samples with the maximum amount of information. Alternatively, the use of similarity measures between the initial structures before the actual running of *ab initio* calculations can be a tool to obtain representative structures of the PES. However, the problem of how to best choose initial structures for NNP generation is still open.

On the other hand, the processing of information by the model can be enhanced to facilitate the convergence of the model, make it more data efficient and reduce the dependency on the initial points. This has been explored for equivariant NNs which complement the description of the interactions in the message step of MPNNs (see Section 3.2). Equivariant NNs have been proven to be very data efficient by obtaining an accuracy comparable to the best NNPs using only a fraction of the data that other methods require.^96^ As a complement to this strategy, it is possible to obtain data efficient models by including more physics-based information which has been proved to perform better than regular approaches for kernel methods.^259^

### 8.4 Reliable active learning and uncertainty quantification

A complete exploration of a PES is a challenging task that most likely can not be done in a single step and depends heavily on the application. Therefore, the improvement of PESs is an active topic of research. Algorithms for systematically improving a training dataset are known as “active learning” techniques. Active learning is closely related to uncertainty quantification of the predictions, which by itself is an active area of research. For NNPs, the most common technique for obtaining the uncertainty is the training of ensembles of NNs which are then averaged for the prediction of identical points. This procedure has a high computationally price because it requires the training and evaluation of several NNPs. As mentioned before, ensemble methods present a clear drawback because their estimated uncertainty can only quantitatively relate to the observed error.^162^

Other methods of uncertainty quantification like Bayesian NNs, which impose a prior distribution to each of the parameters of a NNP are computationally too expensive for practical use.^260^ However, Gaussian processes are a limiting case of Bayesian NNs,^261^ which have been extensively used and applied for the refinement of PESs by means of UQ.^262,263^ Therefore, a combination of NNPs and Gaussian process regression is a promising avenue for UQ in NNPs. Another approach for UQ is single network deterministic methods^163,264^ which make assumptions about the distribution of the data. These methods appear to be a promising alternative to the mentioned problems by obtaining the uncertainty by training and evaluating a single model (see Fig. 6C). However, it should be noted that single network models are strongly influenced by the initial assumptions and it is necessary to calibrate the model beforehand. The need for adjustments is not an exclusive problem of single network models. All the previously described methods require a step of calibration in order to assure that the predicted uncertainties can be related to the observed error. Finally, it should be mentioned that active learning techniques without uncertainty quantification have not been tested.^265^

### 8.5 Extrapolation outside the training set covered

One of the major drawbacks of NNs is their limited capability to extrapolate in general beyond the training data.^266^ For the case of NNPs this means that evaluating energies and forces for structures not covered in the training/validation are likely to lead to a severe breakdown of the model. This weakness stems from the fact that the functional form lacks a physical basis and is a pure mathematical fitting procedure.^267^ This is different for methods such as reproducing kernels (RKHS) and PIPs. RKHS allows to choose kernel functions to follow the physics of the long-range part of the intermolecular interactions.^27,28,268–270^ PIPs make use of Morse variables (*i.e.* internuclear distances are usually transformed to Morse variables) which decay to zero for large distances giving the PES fit a qualitatively correct asymptotic behaviour.^271^ However, to obtain the correct long-range behaviour, PIP PESs often employ switching functions.^33,272^ The inability of extrapolation for NNPs is often revealed at early stages of the NNP generation and can, *e.g.*, be expressed by unphysical short interatomic distances or by a partial or entire fragmentation of the system.^111^ Thus, a possible route for improvement is to include explicit physical knowledge, *e.g.*, on the long-range electrostatic interactions,^51,90,91^ dispersion corrections,^51^ or on nuclear repulsion.^89^ Such extensions are likely to allow extrapolation beyond the training data. Besides the extrapolation in configurational space, the extrapolation and transferability across chemical space is of concern.

### 8.6 Enhancing PESs to higher levels of theory

Transfer learning and Δ-ML is a comparatively new concept for theoretical chemistry and solid evaluations are needed. One of the questions that arises is how to validate the quality of a TL-PES if single point calculations become increasingly expensive. In other words, if the effort to carry out one single point *ab initio* calculation for the HL model required for TL becomes too large, it is preferable to keep this data in the training set for TL instead of using it for testing. This certainly gives rise to the question as to how to probe and validate the NNP for regions that lie outside of the TL data set. One possible strategy to test the improvement of the HL PES with respect to the LL PES is to calculate an observable, compare it to experiment and check for a convergence towards the experiment, as was done by some of us for the determination of tunneling splittings.^193^

Another open question is what the lowest possible level of theory for the LL-model is which still allows reliable TL to a HL-model. The answer to this question will depend on the system and application considered. Ideally, Hartree–Fock calculations would be a suitable surrogate model for TL to CCSD(T) levels of theory, but this needs to be explored for specific systems.^170^

Finally, since the computational cost of the quantum chemical calculations can be appreciable, again the judicious selection of molecular structures for which HL calculations are carried out for TL is crucial. While no simple answer to this question exists as of now, the structures are usually carefully chosen with human intervention. Alternatively, it is conceivable that an approach similar to on-the-fly ML^160,161^ (“on-the-fly TL”) could be used to select data points to include in the TL data set.

### 8.7 Other challenges

Finally, a number of other challenges are briefly summarized. With the ever increasing quality of NNPs a better understanding of the relationship between the accuracy of a NNPs based on reference data for a given quality of the electronic structure and the observables determined from simulations using this PES is required. Ultimately, this requires a direct comparison with experiment. This raises the question whether it is possible to determine the underlying PES from inverting the relationship between observables and interaction potential, *e.g.* by using invertible NNs.^273–275^ Such an inversion has been done successfully for low-dimensional systems. The Rydberg–Klein–Rees (RKR)^276–278^ and rotational RKR (RRKR)^279^ procedures are examples for this. However, for high-dimensional systems, this is a formidable task and will require a large number of high-quality data. With respect to the quality of the trained models, more informative statistical measures should be developed because those used at present often hide poor performance in individual structures.

Another challenge ahead is the seamless integration of NNPs – or ML models in general – into standard MD simulation packages while not compromising their computational efficiency. Further improvements of NN-based interaction potentials can be expected from using physics-informed NNs.^280,281^ Another possibility is to explore the combination of NN-based representations at short range with physics-based long range models based on multipolar and/or polarizable models.

Technically, the question arises how complete descriptors need to be for a comprehensive and accurate representation of the intermolecular interactions *i.e.* what is a meaningful balance between the size of the descriptor(s) and the accuracy of the final model? Additionally, recent advancement in quantum computing technologies provides opportunities to further reduce the computational cost for generating, training and applying NNPs.^282^ Still, whether and how these developments will impact how NNPs evolve and are being used is an open question.

On the more societal side, it is noted that constructing a full-dimensional PES for one given molecule is often a computational investment that requires appreciable resources. Hence, the environmental impact of this should be considered as well.^283–285^ Generally, all ML-based PESs require the *ab initio* computation of information (energies, forces, or both) for thousands of nuclear geometries followed by the training of a model which incurs appreciable environmental cost.††An interesting tool to check the CO_2_ production of your algorithms can be found at: http://www.green-algorithms.org/.

## 9 Conclusion

The field of NNPs has reached a considerable degree of maturity in conceiving PESs that can be used in concrete applications, be it within the exploration of individual structures or in dynamics-based studies. Also due to the tremendous progress in efficiency of electronic structure calculations, it is now possible to determine full-dimensional – not necessarily “global” – potential energy surfaces for medium-sized molecules at levels of theory that allow direct comparison and in some cases even prediction of experimental observables. This, combined with techniques such as transfer learning holds promise to design yet improved PESs.

On the other hand, a rather unexplored facet of NNPs concerns questions about the interpretation of the underlying NN from a chemical perspective, aspects relating to the optimal distribution of reference points including minimizing the number of such calculations, or transferring PESs from one chemical system to a related species without recomputing all reference information afresh. Solutions to these questions will considerably increase the efficiency for conceiving and evaluating NNPs, and improve the prospects for generalizing trained models to broader chemistries and applications.

The present contribution aims at consolidating the available technical approaches, their use in constructing PESs and their application in concrete molecular simulations. It is hoped that this will provide a basis for further development because the prospects of NNPs are bright and the future for them is open.

## Data availability

As this is a perspective article, no primary research results, data, software or code have been included.

## Conflicts of interest

There are no conflicts to declare.

## Supplementary Material
